# Protein disulfide isomerase-9 interacts with the lumenal region of the transmembrane endoplasmic reticulum stress sensor kinase, IRE1, to modulate the unfolded protein response in *Arabidopsis*

**DOI:** 10.3389/fpls.2024.1389658

**Published:** 2024-05-16

**Authors:** Rina Carrillo, Kaela Iwai, Alena Albertson, Gabrielle Dang, David A. Christopher

**Affiliations:** Department of Molecular Biosciences and Bioengineering, University of Hawaii, Honolulu, HI, United States

**Keywords:** disulfide, endoplasmic reticulum stress, proteostasis, unfolded protein response, UPR-regulation

## Abstract

Environmental stressors disrupt secretory protein folding and proteostasis in the endoplasmic reticulum (ER), leading to ER stress. The unfolded protein response (UPR) senses ER stress and restores proteostasis by increasing the expression of ER-resident protein folding chaperones, such as protein disulfide isomerases (PDIs). In plants, the transmembrane ER stress sensor kinase, IRE1, activates the UPR by unconventionally splicing the mRNA encoding the bZIP60 transcription factor, triggering UPR gene transcription. The induced PDIs catalyze disulfide-based polypeptide folding to restore the folding capacity in the ER; however, the substrates with which PDIs interact are largely unknown. Here, we demonstrate that the *Arabidopsis* PDI-M subfamily member, PDI9, modulates the UPR through interaction with IRE1. This PDI9–IRE1 interaction was largely dependent on Cys63 in the first dithiol redox active domain of PDI9, and Cys233 and Cys107 in the ER lumenal domain of IRE1A and IRE1B, respectively. *In vitro* and *in vivo*, PDI9 coimmunoprecipitated with IRE1A and IRE1B. Moreover, the PDI9:RFP and Green Fluorescence Protein (GFP):IRE1 fusions exhibited strong interactions as measured by fluorescence lifetime imaging microscopy-fluorescence resonance energy transfer (FLIM-FRET) when coexpressed in mesophyll protoplasts. The UPR-responsive PDI9 promoter:mCherry reporter and the UPR-dependent splicing of the bZIP60 intron from the mRNA of the 35S::bZIP60-intron:GFP reporter were both significantly induced in the *pdi9* mutants, indicating a derepression and hyperactivation of UPR. The inductions of both reporters were substantially attenuated in the *ire1a–ire1b* mutant. We propose a model in which PDI9 modulates the UPR through two competing activities: secretory protein folding and via interaction with IRE1 to maintain proteostasis in plants.

## 1 Introduction

The endoplasmic reticulum (ER) is the central organelle for the synthesis, proper folding, and post-translational modification of secretory and plasma membrane proteins. In plants, these proteins play major roles in cell wall formation, cell–cell communication in the apoplasm, plant development, and defense and stress responses (Wang et al., 2020). The ER also senses cellular stress to maintain proteostasis when adapting to biotic and abiotic environmental challenges (Yuen et al., 2013). Such disruptive environmental stresses induce the accumulation of unfolded and misfolded proteins, known as ER stress (Angelos et al., 2017; Bao and Howell, 2017). Under prolonged ER stress, the normal protein folding machinery becomes overburdened, such that the rate of protein folding and modification fails to meet the translational output in the ER (Fanata et al., 2013). Perturbation of ER homeostasis and protein folding is associated with various diseases caused by abnormal proteins in mammalian models, including hypoxia, neurodegeneration, cancer, and diabetes. In plants, the disruption of ER homeostasis is associated with poor seed development, misregulation of programmed cell death (Ondzighi et al., 2008), autophagy (Pu and Bassham, 2013; Zhang et al., 2020), light and heat stress (Lu and Christopher, 2008; Deng et al., 2011; Feldeverd et al., 2020), as well as altered protein trafficking (Williams et al., 2014; Korner et al., 2015; Yuen et al., 2017).

The maintenance of protein homeostasis is imperative to preserve individual protein functionality, prevent chronic cellular stress, and prevent the accumulation of unfolded and aberrantly folded proteins, ultimately sustaining plant growth (Okumura et al., 2015; Bao and Howell, 2017; Brandizzi, 2021). As relief, a conserved signaling pathway known as the unfolded protein response (UPR) is activated, which serves as a communication mechanism between the ER and the nucleus to upregulate protein folding enzymes and chaperones to increase the protein folding capacity in the ER (Williams et al., 2014; Shao and Hegde, 2016). In addition, excess misfolded proteins that cannot be folded are proteolytically degraded via ER-associated protein degradation (ERAD; Liu and Howell, 2016) through either the ubiquitin-proteosome pathway (ERAD-I; Book et al., 2010) or the autophagy/lysosome pathway (ERAD-II; Houck et al., 2014). Although the UPR clearly is needed for plant survival, the unabated operation of the UPR can also induce oxidative stress (Ozgur et al., 2014), programmed cell death, and autophagy (Pu and Bassham, 2013; Manghwar and Li, 2022). Thus, the UPR pathway must be tightly regulated in accordance with the protein folding requirements within the ER to protect the cell from dysfunctional signaling or maladaptation to adverse environmental conditions (Williams et al., 2014).

The primary sensor of the UPR in eukaryotes is the inositol-requiring enzyme-1 kinase (IRE1), which detects the presence of the unfolded proteins in the ER and activates the bZIP60 transcription factor that upregulates expression of chaperones and foldases (Lu and Christopher, 2008). IRE1 detects the presence of unfolded proteins through its ER-localized lumenal domain, resulting in dimerization and autophosphorylation of its cytosolic kinase domain and subsequent activation of its ribonuclease (RNase) “splicing” domain (Amin-Wetzel et al., 2018). In plants, activated IRE1 catalyzes the unconventional (cytosolic) splicing of *bZIP60* mRNA, encoding a transcription factor responsible for the activation of UPR target genes such as protein disulfide isomerases (PDIs) and other chaperones (Mishiba et al., 2013). Three IRE1 isoforms have been described in *Arabidopsis*: IRE1A, IRE1B, and IRE1C. The latter lacks an ER lumenal domain but retains cytosolic kinase/RNase activity. IRE1C may play an alternative supportive role in gametogenesis that circumvents ER stress-induced lumenal domain activation (Mishiba et al., 2019).

To assist with protein folding and maintenance of proteostasis in the IRE1 pathway, ER-resident PDIs facilitate the formation, breakage, and rearrangement of disulfide bonds in a variety of client proteins (Yuen et al., 2013). The PDI family is characterized by having one or more catalytic domains sharing sequence homology to thioredoxin (Trx), a 10-kDa enzyme involved in thiol-disulfide redox reactions that contains a conserved CXXC catalytic motif. The cysteine residues within this dithiol active site are responsible for oxidation and reduction to form and break disulfide bonds, respectively, in client proteins (Wilkinson and Gilbert, 2004). The canonical PDI gene (mammalian *PDIA1*) encodes a protein consisting of four sequential domains (a–b–b’–a’). The a and a’ domains possess the catalytic (redox-active) motif sharing homology to thioredoxin, whereas the b and b’ domains are redox-inactive thioredoxin-fold domains (Yuen et al., 2013; Khan et al., 2016).

*Arabidopsis thaliana* encodes 14 PDI-like proteins, including six that share the canonical domain arrangement a–b–b’–a’, and two orthologs of mammalian PDIA6 (PDI9 and PDI10). PDI9 and PD10 comprise a subfamily (M) that exhibits a unique structural arrangement possessing two closely spaced thioredoxin a-type domains while lacking the intervening b-type domains (designated –a–b) (Yuen et al., 2013). We have previously shown that PDI9 catalyzes oxidative protein folding via disulfide bond formation of alkaline phosphatase (Yuen et al., 2013). No other known interactors of PDI9 have been identified. PDI9 has been shown to play an important role in the ER stress response and the UPR in *Arabidopsis* (Lu and Christopher, 2008; Feldeverd et al., 2020). *PDI9* gene expression is highly upregulated in response to chemically induced UPR and decreased in the *bZIP60* mutant, suggesting *PDI9* as an IRE1-mediated UPR target gene (Lu and Christopher, 2008). Mammalian models also suggest that IRE1α interacts with the PDI9 ortholog, PDIA6, through a critical disulfide bond involving Cys148 in the lumenal domain of IRE1α to limit the duration of UPR signaling (Eletto et al., 2014). We recently showed that PDI9 has a protective role in maintaining pollen development under heat stress, with disturbed pollen exine biogenesis in the *pdi9-1* and *pdi9–pdi10* mutants (Feldeverd et al., 2020). Excessive heat is known to induce ER stress and the UPR (Deng et al., 2011), further supporting the role of PDI9 in maintaining cellular homeostasis under stress (Feldeverd et al., 2020). However, the mechanism by which PDI9 is involved in ER stress and the UPR pathway in plants, as well as any potential interactors such as IRE1, are poorly understood.

To enhance our understanding of these processes, here we present multiple lines of evidence that show *Arabidopsis* PDI9 interacts with the lumenal domain of IRE1 in the ER to modulate the UPR in a manner to protect and maintain proteostasis. These results highlight the need for a better understanding of the intricate mechanisms by which plants mitigate ER stress-inducing events such as heat and promote future agricultural advancements that may improve plant tolerance to stress.

## 2 Materials and methods

### 2.1 Plant materials, growth conditions, mutant analysis, and verification

*Arabidopsis* seeds were germinated vertically on 0.5× Linsmaier and Skoog (LS) medium containing 1.5% (w/v) sucrose and solidified with 0.8% (w/v) gellan gum (Gelrite) and transferred at 1–2 weeks after germination to pots containing Fafard Super Fine Germinating Mix (Sun Gro Horticulture Inc., Agawam, MA, USA) supplemented with 0.5× Miracle-Gro All Purpose Plant Food (The Scotts Miracle-Gro Co., Marysville, OH, USA). Plants were grown at 22°C (for seedlings) and 25°C (for soil-grown plants) under a long-day photoperiod (16-h light, 8-h dark cycle). The PDI9 (At2g32920) and PDI10 (At1g04980) genes were previously characterized (Lu and Christopher, 2008; Feldeverd et al., 2020). Seeds of WT *Arabidopsis* (Columbia, Col-0) and homozygous T-DNA insertion lines *pdi9-1* (WiscDsLox445A08, progeny line CS864623), *pdi10-1* (SALK_206219C), *ire1a* (SALK_002316), and *ire1b* (SAIL_238_F07) were obtained from the *Arabidopsis* Biological Resource Center (ABRC). The thorough characterization of the T-DNA mapping and verification of the homozygosity for the *pdi9-1*, *pdi9-2*, *pdi10-1*, and *pdi9–pdi10* mutants were described in Feldeverd et al. (2020). The *IRE1A* (AT2G17520) and *IRE1B* (AT5G24360) genes contain seven and six exons, respectively. The mapping details for *ire1a* and *ire1b* were also reported by Lu and Christopher (2008) and Deng et al., (2011), respectively. The *ire1a–ire1b* double mutant was created by crossing *ire1a* × *ire1b*, and double homozygous mutant progeny in the F3 generation were confirmed by PCR. Genotyping of the T-DNA insertion mutants was done by extracting genomic DNA (Klimyuk et al., 1993) and PCR verification (MyFi Mix; Bioline) using T-DNA- and gene-specific primers (**Supplementary Figure S9**, primer table). The T-DNA insertion for *ire1a* has been mapped to intron 5, and that for *ire1b* is located in intron 4 (**Supplementary Figure S10**). Genotyping at the *IRE1A* locus was performed using primers SALK_002316_ire1a-3-RP and SALK_002316_ire1a-3-LP to detect the WT allele and primers LBa1 and SALK_002316_ire1a-3-RP to detect *ire1a*. Genotyping at the *IRE1B* locus was performed using primers IRE1B-Int2-F and IRE1B-Int4-R to detect the WT allele and primers SAIL_LB2 and SAIL_238_F07_RP to detect *ire1b*. The *ire1A–ire1B* double-mutant was verified by PCR (35 cycles of 98°C for 15 s, 60°C for 30 s, and 72°C for 1 min), as shown in **Supplementary Figure S10**.

### 2.2 Generation of constructs for transient expression assays in *Arabidopsis* protoplasts

The GFP referred to in all of the experiments is eGFP (S65T), which is the main source construct for GFP as described (Yuen et al., 2013). To generate the GFP : IRE1A construct, the CaMV 35S promoter was PCR-amplified from pCAMBIA1302 with primers engineered with *Kpn*I and *Xho*I restriction sites and was ligated into the corresponding sites of pBluescript KS+. The IRE1A signal peptide, including a portion of the 5′-UTR, was PCR-amplified from Col-0 genomic DNA and ligated into *Xho*I and *Cla*I restriction sites. GFP was then PCR-amplified from the previously reported construct, PDI9:GFP-KDEL (Yuen et al., 2013), using primers engineered with *Cla*I and *Xma*I restriction sites. The mature polypeptide region of IRE1A (At2g17520) was PCR-amplified from Col-0 genomic DNA using primers engineered with *Xma*I and *Bam*HI restriction sites and inserted into the intermediate plasmid from above. The nopaline synthase (nos) 3′-UTR fragment was PCR-amplified from the previously cloned GFP construct (Yuen et al., 2013) with flanking *Bam*HI and *Not*I restriction sites and inserted into the intermediate plasmid to yield the final construct, GFP : IRE1A.

To generate the GFP : IRE1B construct, a portion of the IRE1B 5’UTR (At5g24360), including the signal peptide sequence, was PCR-amplified from Col-0 genomic DNA and ligated into the *Xho*I and *Cla*I sites in the 35S promoter-pBluescript KS+ intermediate plasmid (described above). The GFP coding sequence was PCR-amplified from the previously cloned GFP vector (Yuen et al., 2013) using primers engineered with *Cla*I and *Xma*I sites and ligated into the respective sites in the intermediate plasmid from above. The IRE1B mature polypeptide region was PCR-amplified from Col-0 gDNA with *Xma*I and *Not*I restriction sites, fused to the nopaline synthase (nos) 3′-UTR derived from a previous construct (Yuen et al., 2013) with flanking *Not*I and *Sac*I restriction sites, and ligated into the intermediate plasmid to yield the final GFP : IRE1B construct.

For generation of the PDI9:mCherry-KDEL construct, the previously described PDI9:GFP-KDEL (Yuen et al., 2013) was digested using two subsequent double digestion reactions with *Xma*I/*Bst*EII and *Xma*I/*Bam*HI, followed by ligation with the mCherry insert amplified from the ER-mCherry marker with corresponding *Xma*I and *Bam*HI restriction sites. For the construction of the mCherry control vector, the mCherry fragment was amplified from PDI9:mCherry-KDEL using primers engineered with *Xho*I and *Bam*HI restriction sites and ligated into the plasmid template described above containing the 35S promoter. The 3′-UTR nos terminator sequence was amplified from the main GFP construct and inserted between *Bam*HI and *Not*I restriction sites to yield the final construct.

The FRET positive control dual fluorescent protein fusion construct, pBL(35S:GFP-mCherry) (referred to hereon as GFP:mCherry), was created by replacing the γTIP coding sequence cassette between the *Spe*I and *Bam*HI sites of plasmid pBL(35S:γTIP-mCherry) (Yuen et al., 2013) with an *Spe*I/*Bam*HI fragment encoding GFP. The GFP coding sequence was generated by PCR using the primers GFP(*Spe*)F and GFP(*Bam*HI*Xma*I)R (**Supplementary Figure S9**), using the plasmid pBL-GFP (Yuen et al., 2013) as a template.

The construct encoding the hemagglutinin (HA) epitope-tagged PDI9 protein, HA : PDI9, was generated for co-I.P. in transfected protoplast samples using pBluescript KS+. The nopaline synthase (nos) 3′-UTR fragment was PCR-amplified from the PDI9:mCherry-KDEL construct with flanking *Not*I and *Sac*I restriction sites and ligated into the respective restriction sites in the empty vector. The CaMV 35S promoter sequence was PCR-amplified from the plasmid, PDI9:mCherry-KDEL, using primers engineered with *Eco*RI and *Kpn*I and ligated into the intermediate plasmid from above. The PDI9 signal peptide sequence (including a portion of the 5’ UTR) and the HA tag were PCR-amplified from Col-0 genomic DNA using primers engineered with *Kpn*I and *Bam*HI restriction sites (**Supplementary Figure S9**) and inserted into the corresponding restriction sites of the developing vector. The mature polypeptide region of PDI9 was then amplified from Col-0 genomic DNA with flanking *Bam*HI and *Not*I restriction sites and inserted into the respective sites of the intermediate plasmid from above to yield the final construct, HA : PDI9.

The PDI9-promoter:mCherry-KDEL reporter construct was generated by PCR-amplifying the 5′-flanking sequences approximately 2.7 kb upstream of the start codons of PDI9 (containing the promoter regions) from *Arabidopsis* Col-0 genomic, using primers engineered with *Kpn*I and *Xho*I restriction sites (**Supplementary Figure S9**), and inserted into the respective sites in the mCherry control vector described above. Generation of the 35S::*bZIP60* intron:GFP construct was previously described by Carrillo and Christopher (2022). To confirm transfection efficiencies between protoplast cells expressing varying fluorescence intensities of the PDI9-promoter:mCherry-KDEL construct, cells were cotransfected with the GFP control.

### 2.3 Transient expression assay in *Arabidopsis* protoplasts

Protoplast isolation and transfection were performed using the Tape-*Arabidopsis* Sandwich protocol, as described by Wu et al. (2009) and as modified by Carrillo and Christopher (2022). The transfected protoplasts were incubated in the light at room temperature for 16–18 h before being examined using a Leica TCS SP8 laser scanning confocal microscope at the Biological Electron Microscope Facility (University of Hawaii at Manoa, Honolulu, HI, USA). The excitation/emission filters utilized for fluorescence detection were 488/505–525 nm for GFP and 543/585–615 nm for mCherry. For chemical induction of ER stress and the UPR in protoplasts, samples were inoculated with 2 mM dithiothreitol (DTT) from a 1-M DTT stock (Roche Applied Science, Indianapolis, IN, USA) and incubated for 3 h prior to visualization by scanning confocal microscopy.

### 2.4 Fluorescence lifetime imaging microscopy-fluorescence resonance energy transfer analysis

FLIM for FRET detection was performed using a Leica TCS SP8 confocal laser scanning microscope at the Biological Electron Microscope Facility (University of Hawaii at Manoa, Honolulu, HI). A diode pulsed laser was used to excite GFP, and the emission from 488 nm was collected in 520 pixel × 520 pixel mode. The GFP lifetime was determined from the fluorescence decay curve per pixel, using a photon count rate of 1 photon per laser pulse. Photon counts were accumulated by line repetition to optimize the setting for live protoplasts. The average lifetime in the selected region(s) of interest (ROI) within each of the protoplast cells examined was analyzed with the FLIM pixels binned to select an optimal total photon count > 1,000. The ROIs containing GFP fluorescence (GFP : IRE1A, GFP : IRE1B, GFP, or GFP:mCherry) in all single- and dual-expressed (with PDI9:mCherry-KDEL or ER:mCherry) protoplast samples were selected for average GFP lifetime values. FRET efficiency (*E*) was calculated for each ROI using the following formula:


$$E=\;\left(1-\tau_{i}/\tau_{0}\right)\;\times \;100\%$$

Where τ*_i_* is the mean lifetime for that ROI, and τ_0_ is the average lifetime of all ROIs in GFP : IRE1A and GFP : IRE1B single-transfected samples (not expressing detectable mCherry). At least 20 cells were analyzed for each sample, and average GFP lifetime values were obtained over two independent experiments.

### 2.5 Coimmunoprecipitation in *Arabidopsis* protoplasts

Protoplast isolation and transfections were performed using the Tape-*Arabidopsis* Sandwich protocol (described above) and modified for co-I.P. using a protocol adapted from Shan et al. (2008) and Carrillo et al. (2021). Protoplasts isolated from 4-week-old Col-0 plants were cotransfected with the constructs HAPDI9 and GFP : IRE1A or GFP : IRE1B. Single-construct transfections were prepared as controls. Sample transfection volumes were scaled fivefold to account for a 1-mL final transfection volume per sample (i.e., 150 µg of pDNA per construct mixed with 1 mL of protoplasts in MMg solution and an equal volume of 40% PEG). All subsequent washes were also scaled up fivefold (i.e., 5 mL of washes in W5 solution), and the transfected protoplasts were incubated at RT for 16–18 h prior to protein extraction.

The 1-mL transfected protoplast samples were centrifuged at 100×*g* for 5 min, and the W5 solution was discarded. The pelleted protoplasts were vortexed vigorously for 30 s with 150 µL of protein extraction buffer (nonreducing 10 mM HEPES at pH 7.5, 100 mM NaCl, 1 mM EDTA, 10% glycerol, 0.5% Triton X-100, and 1 mM PMSF) and centrifuged at 13,000 rpm for 10 min at 4°C. The supernatant was collected, and 100 µg of total protein per lysate was incubated with 50 µL of anti-HA magnetic beads for 3 h at 4°C on a rotary shaker (20 rpm). The beads were collected on a magnetic stand using the Pierce HA Tag Magnetic I.P./Co-I.P. kit (Thermo Fisher Scientific, Waltham, MA, USA). Proteins isolated by co-I.P. were separated by reducing SDS-PAGE and analyzed by Western blot with a monoclonal anti-HA antibody (Sigma-Aldrich, St. Louis, MO, USA) or polyclonal anti-GFP antibody (Molecular Probes, San Jose, CA, USA) at 1:2,000 dilution, with a secondary goat antimouse (Advansta Inc., San Jose, CA, USA) or goat-antirabbit (Advansta Inc., San Jose, CA, USA) IgG HRP conjugate antibody, respectfully.

### 2.6 Generation of the PDI9 and IRE1 constructs for *in vitro* coimmunoprecipitation

Constructs for heterologous expression of *Arabidopsis* PDI9 and IRE1A or IRE1B in *Escherichia coli* were generated in the vector pETDUET-1 (Novagen, 2011), designed for the simultaneous coexpression of the two genes. The signal peptides were omitted for PDI9, IRE1A, and IRE1B cDNAs to enable protein expression in the cytoplasm of *E. coli*. The mature protein coding sequence for PDI9 cDNA was first amplified by RT-PCR from *Arabidopsis* Col-0 WT total RNA (isolated using the NucleoSpin RNA Plant Kit, Macherey-Nagel Inc., Duren, Germany), using primers engineered with *Bam*HI and *Not*I restriction sites and inserted into corresponding sites in the pETDUET-1 vector MCS1, fusing it in-frame to the built-in 6×His tag. The coding sequence for the N-terminal lumenal (ER-sensor) domain (LD) of the IRE1A cDNA was amplified by RT-PCR using primers containing *Kpn*I and *Pac*I restriction sites and an internal Strep-tag. This truncated IRE1A fragment was ligated into the same restriction sites in the pETDUET MCS2, thus fusing it in the frame to the Strep-tag to generate the final HisPDI9-StrepIRE1A_LD_ construct.

To make the HisPDI9-StrepIRE1B_LD_ construct, the N-terminal LD of the IRE1B cDNA was amplified by RT-PCR as described above for IRE1A. RT-PCR used modified primers containing a Strep-tag and *Kpn*I and *Pac*I restriction sites. The digested fragment was ligated into *Kpn*I and *Pac*I sites in the MCS2 of the pETDUET-1 containing the PDI9 cDNA. This produced the final HisPDI9-StrepIRE1B_LD_ construct. Single-insert vectors were generated as controls that contained only the HisPDI9 StrepIRE1A_LD_ or StrepIRE1B_LD_ coding regions.

### 2.7 Construction of site-specific mutants

The second cysteine residue in the two thioredoxin active sites of PDI9 (positions 63 and 195) and the two cysteine residues in the ER lumenal domain of IRE1A (positions 233 and 257) and IRE1B (positions 107 and 222) were individually mutated to alanine (Kersteen et al., 2005). The previously designed constructs described above were used as template plasmids for generating the respective site-specific mutations for use in both *in vivo* protoplast fluorescence lifetime imaging microscopy-fluorescence resonance energy transfer (FLIM-FRET) and *in vitro* coimmunoprecipitation assays. Primers were designed to contain the desired mutation, and mutagenesis was confirmed by DNA sequencing (GeneWiz Inc., Plainfield, NJ, USA). A summary of primers used in this study is shown in **Supplementary Figure S9**. For *in vitro* co-I.P. assays, the following mutants were generated in the pETDUET-1 expression vector: HisPDI9(C63A)-StrepIRE1A_LD_, HisPDI9-StrepIRE1A_LD_ (C233A), HisPDI9-StrepIRE1A_LD_ (C257A), HisPDI9(C63A)-StrepIRE1B_LD_, HisPDI9(C195A)-StrepIRE1B_LD_, HisPDI9-StrepIRE1B_LD_(C107A), and HisPDI9-StrepIRE1B_LD_(C222A), including their associated single-vector controls. For *in vivo* protoplast expression assays, the following mutants were generated in the pBluescript KS+ expression vector: PDI9(C63A):mCherry-KDEL, PDI9(C195A):mCherry-KDEL, GFP : IRE1A(C233A), GFP : IRE1A(C257A), GFP : IRE1B(C107A), and GFP : IRE1B(C222A).

### 2.8 Expression of recombinant proteins in *E. coli*

To promote the formation of disulfide bonds in the cytoplasm of *E. coli*, the expression host Rosetta-gami 2 (DE3) was used, which carries the glutathione reductase (*gor*) and thioredoxin reductase (*trxB*) mutations (Novagen, 2011). The *E. coli* Rosetta-gami 2 (DE3) was transformed with the following pETDUET-1-cloned plasmids: HisPDI9-StrepIRE1A_LD_, HisPDI9-StrepIRE1B_LD_, HisPDI9, StrepIRE1A_LD_, and StrepIRE1B_LD_, including all aforementioned mutant constructs containing the specific cysteine to alanine substitution. Transformed cells were grown to approximately OD600nm = 0.5–0.6 and induced with 0.2 mM IPTG for 3 h at 30°C. After induction, cells were harvested for extraction of soluble proteins under nonreducing conditions using the BugBuster Protein Extraction Reagent (EMD Millipore, Burlington, MA, USA) supplemented with phenylmethylsulfonyl fluoride (PMSF), benzonase nuclease, and rLysozyme (EMD Millipore, Burlington, MA, USA).

### 2.9 Ni-NTA-His Bind affinity chromatography

Approximately 100 µg of total protein from each of the dual-expressed and single-vector lysates described above were incubated with 100 µL of the nickel-nitrilotriacetic acid (Ni-NTA) His Bind resin slurry (EMD Millipore) under nonreducing conditions and incubated at 4°C on a rotary shaker (20 rpm) for 3 h. The protein–resin mixture was loaded onto a 1-mL Pierce Spin Column (Thermo Fisher Scientific) and centrifuged for 1 min at 1,000×*g* to collect the flow through. The resin was washed twice with 400 µL 1× Ni-NTA wash buffer and eluted with 30 µL 1× Ni-NTA elution buffer. A total of three elutions were performed with a 1-min incubation with the protein–resin mixture. All fractions (unbound flow through, washes, and elutions) were analyzed by SDS-PAGE under reducing conditions as previously described (Yuen et al., 2017).

A 20-µL aliquot of each fraction collected from the Ni-NTA His binding affinity columns was loaded on 10% polyacrylamide gels. Proteins were separated by SDS-PAGE and electrotransferred to Amersham Protran nitrocellulose membranes (GE Healthcare Life Sciences, Milwaukee, WI, USA). Immunoblot analyses were performed using a monoclonal anti-6× His antibody (Sigma-Aldrich, St. Louis, MO, USA) at 1:2,000 dilution or monoclonal anti-Strep antibody (EMD Millipore, Burlington, MA, USA) at 1:1,000 dilution and a horse-radish peroxidase (HRP)-conjugated antimouse IgG secondary antibody (Advansta Inc., San Jose, CA, USA) at 1:20,000 or 1:10,000 dilutions, respectively. Chemiluminescent detection of HRP was done using the Advansta WesternBright ECL HRP substrate (Advansta Inc., USA).

### 2.10 Quantitative RT-PCR of UPR genes in DTT-treated protoplasts

To analyze transcript levels of UPR marker genes, quantitative PCR (qPCR) was done on RNA from *Arabidopsis* protoplasts from the WT, *pdi9-1*, and *pdi9–pdi10* genotypes, using a protocol adapted from Carrillo et al. (2022). Total RNA from 4-week-old *Arabidopsis* protoplasts (*n* = 3) was extracted using the NucleoSpin RNA Plant, Mini Kit (Macherey-Nagel, Duren, Germany). Protoplasts from each genotype were resuspended in RAP lysis buffer with mercaptoethanol (1% v/v) and immediately vortexed to mix. Respective samples treated with 2 mM DTT were incubated for 3 h prior to the harvesting of cells and subsequent RNA extraction.

The qPCR primers were designed using Primer3 (v. 0.4.0) to identify amplicons around 200 bp with a melting point of around 63°C. The synthesis of first-strand cDNA qPCR was prepared at the University of Hawaii Cancer Center Genomics and Bioinformatics Shared Resource (UHCC GBSR) facility. For qPCR, cDNAs were diluted to 10 ng/µL using water, and 1 µL (10 ng) per qPCR reaction was used. PCR reactions were done under the following cycling conditions: 15 s denaturation at 94°C, 30 s annealing at 60°C, and 30 s extension at 72°C for a total of 40 cycles. Gene expression analysis was done using Power SYBR Green PCR Master Mix (Thermo Fisher Scientific), and each 10 µL reaction consisted of 5 µL of Master Mix, 0.41 µL of each forward and reverse primer (10 µM), 3.18 µL of water, and 1 µL of cDNA. A melting curve was generated to verify sequence-specific amplification of PCR products. The genes of interest were prepared in triplicate, and the expression level was determined using cycle threshold (Ct) values with a standard curve after normalization with the housekeeping gene, actin. Fold change was calculated by this method for each gene, and the data are shown as mean with standard deviations. Significance was calculated using one-way analysis of variance (ANOVA) followed by Tukey’s test to compare differences between multiple means and determine significance at *p*-values< 0.01 and< 0.05. Primers used for amplification of *PDI9* (PDI9-F, PDI9-R), *bZIP60t* (bZIP60-F, bZIP60-R), *bZIP60s* (bZIP60-F, bZIP60s-R), *BiP2* (BiP2-F, BiP2-R), and *actin* (Actin2-F, Actin2-R) transcripts are listed in **Supplementary Figure S9**. Prior to qPCR, RNA samples were analyzed on denaturing RNA gels and by standard RT-PCR to verify the RNA quality and expression of genes in all of the samples.

### 2.11 Total fluorescence quantification of protoplast cells

To quantify total fluorescence levels in *Arabidopsis* protoplasts from each genotype transiently expressing either the PDI9-promoter:mCherry or 35S::*bZIP60* intron:GFP, protoplasts were transfected as described above, and DTT was added to a final concentration of 2 mM where indicated. Protoplasts were imaged on a single plane at ×40 magnification (512 by 512 frame resolution) using a Leica TCS SP8 laser scanning confocal microscope (Leica Inc., Teaneck, NJ, USA). All images were imported as Tif files in ImageJ software (NIH) for quantification of total cell fluorescence. Individual cells were selected and outlined using the “freehand selection” tool and measured for area, integrated density, and mean gray value. Background intensity values were measured by selecting five random areas in the image that do not contain cell fluorescence. Corrected total cell fluorescence (CTCF) values were calculated for each cell using the following equation:


CTCF=Integrated intensity−(Area of cell × Mean fluorescence of background readings)

an CTCF values for mCherry and GFP lifetimes were plotted for each reporter construct expressed in the protoplasts of the Col-0, *pdi9-1*, *pdi9-2*, and *pdi9–pdi10* genotypes with 30–50 cells analyzed for each construct in two independent experiments. Significance was calculated using the two-way ANOVA followed by the Tukey’s test to compare differences between multiple means and determine significance at *p*-values< 0.01.

## 3 Results

### 3.1 IRE1A colocalizes and interacts with PDI9 in the ER in *Arabidopsis*

**Figure 1:**
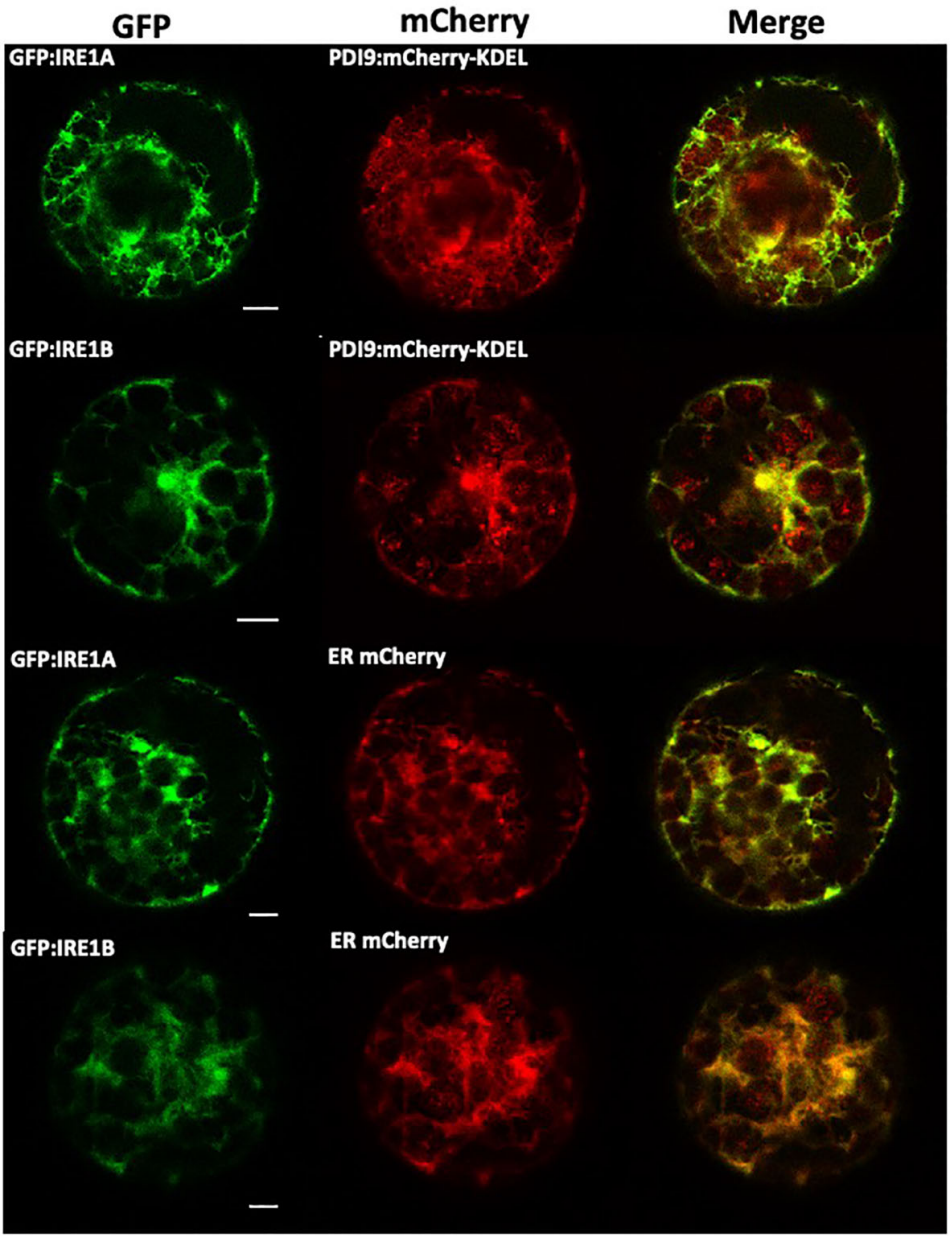
Colocalization of PDI9-mCherry-KDEL and GFP : IRE1 fusions with the ER marker in *Arabidopsis* leaf mesophyll protoplasts. The following constructs were transiently expressed in the protoplasts as indicated: GFP : IRE1A, GFP : IRE1B, PDI9:mCherry-KDEL, and the ER marker, ER:mCherry (Yuen et al., 2013). Representative single confocal planes are shown (from left to right): The GFP signal is shown in column 1. The mCherry signal is shown in column 2 and a merge of two channels in column 3.

Of the *Arabidopsis* PDI family, the PDI9 protein sequence is most similar to the human ortholog, PDIA6, sharing 42% identity and 61% similarity (**Supplementary Figure S1**). Since PDIA6 interacts with IRE1A in the ER of mammals (Eletto et al., 2014), we tested if PDI9 also interacted with IRE1A and IRE1B in the ER of *Arabidopsis*. First, we examined whether PDI9 colocalizes with IRE1A and IRE1B in the ER, as PDI9 is well-documented to be a resident of the ER lumen (Yuen et al., 2013; Feldeverd et al., 2020). Colocalization was tested by transiently expressing the corresponding PDI9 and IRE1 proteins fused to either mCherry or GFP reporters, respectively, in mesophyll protoplasts (**Figure 1**). When GFP : IRE1A or GFP : IRE1B were coexpressed with PDI9:mCherry-KDEL, and examined by confocal laser scanning microscopy, significant colocalization of the fluorescent fusions was observed in the ER (**Figure 1**). Localization patterns were similar when compared with the ER marker, ER:mCherry.

Since our transient expression system correctly detects PDI9 with IRE1A and IRE1B in the ER, the next step was to determine if PDI9 interacted with either IRE1A or IRE1B. The following two methods were chosen for this determination: (a) *in vivo* fluorescence resonance energy transfer (FRET) analysis using fluorescence lifetime imaging microscopy (FLIM) of reporter fusions; and (b) *in vitro* coimmunoprecipitation. For the FLIM-FRET, the PDI9:mCherry-KDEL and GFP : IRE1 fusions were dually expressed in *Arabidopsis* protoplasts, and GFP lifetime decay values were measured in regions of colocalization relative to that of various control samples, which included expressing GFP : IRE1 alone and GFP : IRE1 with mCherry alone. In addition, the dimer, GFP:mCherry direct fusion, was created for this study as a positive control to indicate maximum GFP quenching for comparison with the test samples.

**Figure 2:**
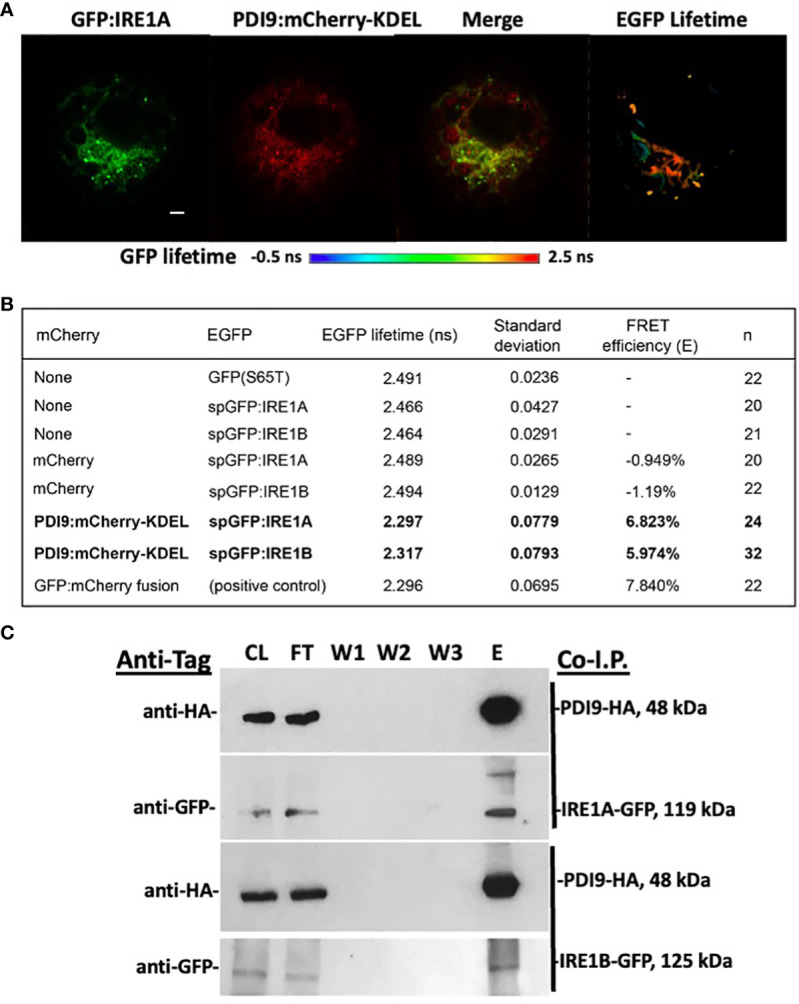
*In vivo* FLIM-FRET analysis between GFP : IRE1 and PDI9:mCherry-KDEL in *Arabidopsis* leaf protoplasts. **(A)** Representative confocal images of a protoplast cell co-expressing GFP : IRE1A and PDI9:mCherry-KDEL. The GFP signal is shown (left), as is the mCherry signal (middle), and a merge of both signals (right). A fluorescence lifetime image bar is also shown under the protoplast images representing GFP lifetimes by a color scale depicted (−0.5 ns to 2.5 ns). FLIM pixels were filtered to show total photon counts >1,000. **(B)** GFP fluorescent lifetime measurements of the GFP : IRE1 and PDI9:mCherry-KDEL interactions, including standard deviation and calculated FRET efficiencies (E). The positive control direct fusion, GFP-mCherry, indicated maximum FRET quenching. **(C)** *In vivo* coimmunoprecipitation of HA-PDI9 co-expressed with GFP : IRE1A or GFP : IRE1B in *Arabidopsis* protoplasts using anti-HA magnetic beads followed by immunoblot analysis with indicated antibodies. CL, crude lysate; FT, flow through; W1, wash 1; W2, wash 2; E, elution.

The mean lifetime of GFP fluorescence (GFP : IRE1A and GFP : IRE1B) in cells coexpressed with PDI9:mCherry-KDEL was significantly diminished relative to GFP : IRE1 control cells (**Figures 2A, B**). This reduction in GFP lifetime indicated that quenching occurred for the fluorescence of the GFP donor in the presence of PDI9-mCherry. Quenching of the donor lifetime was not observed when GFP : IRE1 fusions were coexpressed with the negative control, the mCherry construct (lacking PDI9) (**Figure 2B**). Furthermore, no statistical difference in GFP lifetime values was observed between cells coexpressing GFP : IRE1 fusions and PDI9:mCherry-KDEL and cells expressing the positive control fusion, GFP:mCherry. Therefore, in cells coexpressing either GFP : IRE1A or GFP : IRE1B with PDI9:mCherry-KDEL, the decreases in GFP lifetime values were due to the interaction of IRE1 (A and B) with PDI9.

FRET efficiency (*E*) values were calculated (**Figure 2B**) for each of the dual-expressed samples based on their mean GFP lifetime values relative to the GFP : IRE1 baseline values (single-transfected control samples). The FRET efficiency between the PDI9:mCherry-KDEL and GFP : IRE1A donor:acceptor pair yielded 6.82%, while that for the PDI9:mCherry-KDEL and GFP : IRE1B pair yielded 5.97% (**Figure 2B**). These values were 1% to 1.8% less than, but comparable to, the FRET efficiency of the positive control, GFP:mCherry direct fusion, at 7.84%. A negative value was obtained for GFP samples coexpressed with mCherry alone, as the mean GFP lifetime values were slightly higher than the single-transfected controls (**Figure 2B**).

To further test if the interaction occurred between full-length IRE1 and PDI9 *in vivo*, anti-HA co-I.P. assays were conducted on extracts from *Arabidopsis* protoplasts coexpressing HA-tagged PDI9 (HA : PDI9) with either GFP : IRE1A or GFP : IRE1B (**Figure 2C**). Both HA : PDI9 and GFP : IRE1 signals were detected together in the eluates of protein extracts from cotransfected protoplasts containing HA : PDI9 coexpressed with either the GFP : IRE1A or GFP : IRE1B fusions (**Figure 2C**), relative to single vector controls (**Supplementary Figure S2**). Bands of approximately 119 kDa and 125 kDa in size were detected correlating to GFP : IRE1A and GFP : IRE1B fusion proteins, respectively, using an anti-GFP antiserum on immunoblots of the eluates. The 48-kDa HA : PDI9 was also detected in the same fraction, suggesting corelease of GFP : IRE1A and GFP : IRE1B from the column and thus binding between the two putative interactors, which are the HA : PDI9 with the GFP : IRE1 fusions (**Figure 2C**). These results, together with the FLIM-FRET analysis (**Figures 2A, B**), indicated that PDI9 interacted with both IRE1A and IRE1B *in vivo* in *Arabidopsis*.

### 3.2 The PDI9 and IRE1 interaction is largely dependent on the thioredoxin active site cysteine, Cys63, of PDI9 with the ER lumenal domain cysteines of IRE1

**Figure 3:**
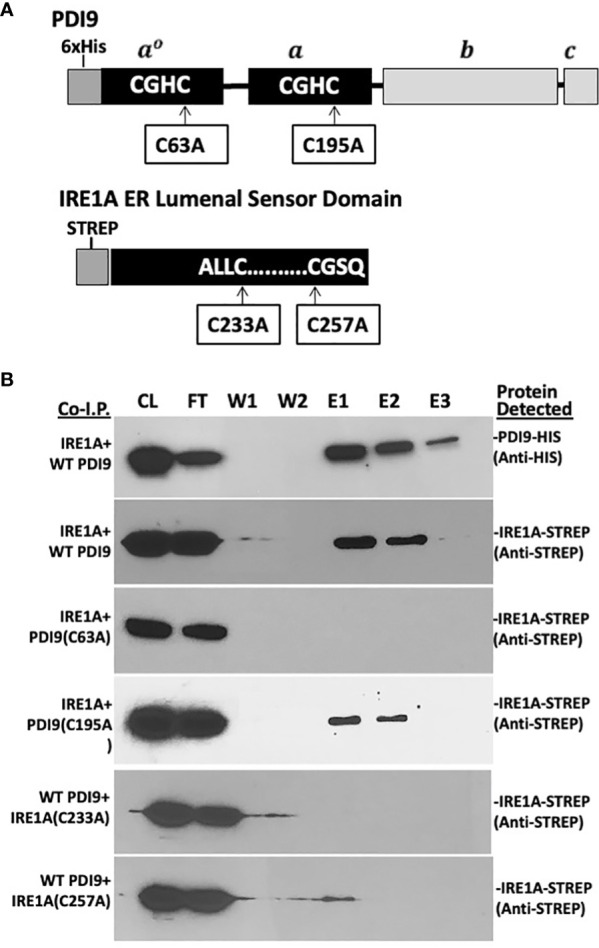
*In vitro* co-I.P. analysis detects the interaction of PDI9 with the IRE1A lumenal sensor domain: assessing the impact of key cysteine mutations on the interaction. **(A)** Location of the cysteine residues selected for site-specific mutagenesis of the respective proteins PDI9(C63A), PDI9(C195A), IRE1A(C233A), and IRE1A(C257A). **(B)** The IRE1A lumenal domain interacts with unmodified PDI9 in *Escherichia coli*. HisPDI9 and StrepIRE1A were coexpressed in *E*. *coli* cells. Affinity chromatography was performed using HisPDI9 as bait on a Ni-NTA His-binding column with the co-I.P. protein pairings (denoted on the left of the blot image). Associated proteins (including mutant versions) were detected by immunoblot analysis using either the anti-His or anti-Strep antibodies (labels to the right of the blot). CL, crude lysate; FT, flow through; W1, wash 1; W2, wash 2; E1, elution 1; E2, elution 2; E3, elution 3.

**Figure 4:**
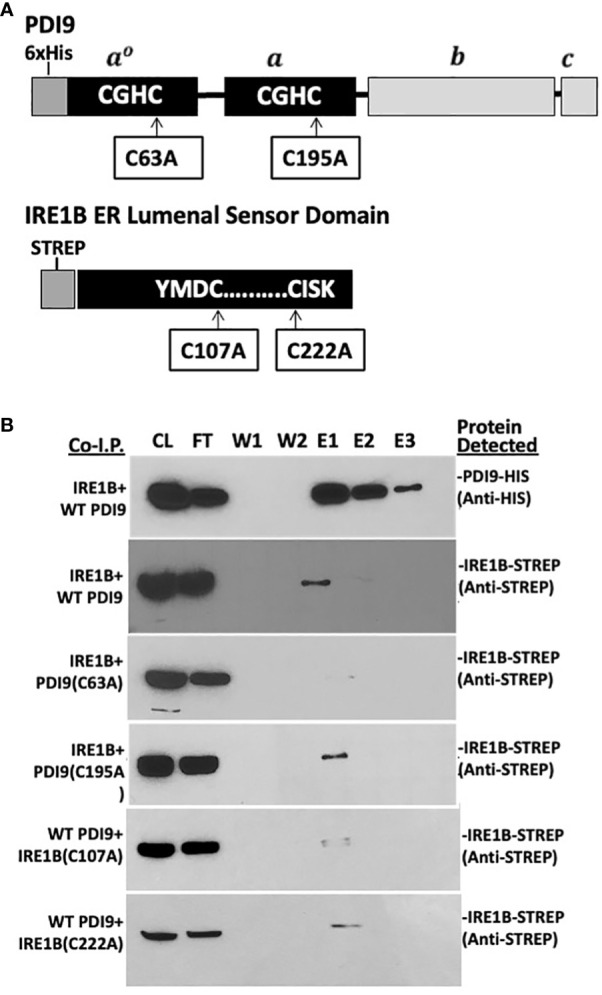
*In vitro* co-I.P. analysis detects the interaction of the PDI9 with the IRE1B lumenal sensor domain: assessing the impact of key cysteine mutations on the interaction. **(A)** Location of the cysteine residues selected for site-specific mutagenesis of the respective proteins PDI9(C63A), PDI9(C195A), IRE1B(C107A), and IRE1B(C222A). **(B)** The IRE1B lumenal domain interacts with unmodified PDI9 in *E*. *coli*. His-PDI9 and Strep-IRE1B were coexpressed in *E*. *coli* cells. Affinity chromatography was performed using His-PDI9 as bait on a Ni-NTA His-binding column with the co-I.P. protein pairings (denoted on the left of the blot image). Associated proteins (including mutant versions) were detected by immunoblot analysis using either the anti-His or anti-Strep antibodies (labels to the right of the blot). CL, crude lysate; FT, flow through; W1, wash 1; W2, wash 2; E1, elution 1; E2, elution 2; E3, elution 3.

To further examine the mechanism of the PDI9-IRE1 protein–protein interactions, a series of co-I.P. experiments were conducted in *E. coli*. The recombinantly expressed WT HisPDI9 and the ER lumenal/sensor domains of IRE1A and IRE1B (fused to the Strep-tag) were used (**Figures 3A, B**, **4A, B**). To obtain the ER lumenal/sensor domains, the mature polypeptide sequences of the IRE1A and IRE1B Strep fusions were truncated by removing the cytoplasmic TMD and kinase domains (referred to as StrepIRE1A_LD_ and StrepIRE1B_LD_). This truncation also prevented protein aggregation when expressed in *E. coli* cells because the presence of the insoluble TMD shunted the foreign IRE protein into inclusion bodies. Expressing the specific ER lumenal/sensor domains overcame this problem.

Both the 36-kDa StrepIRE1A_LD_ and the 40-kDa StrepIRE1B_LD_ proteins were enriched in the HisPDI9 fractions that were eluted from the nickel column, as revealed using the anti-Strep antibody in the immunoblot analysis (**Figures 3B**, **4B**). The wash-resistant presence of StrepIRE1A_LD_ and StrepIRE1B_LD_ only in the eluted HisPDI9 samples indicated the binding of IRE1A_LD_ and IRE1B_LD_ to PDI9. Based on the eluted band intensity, the interaction of IRE1A_LD_ with PDI9 (**Figure 3B**) was stronger than the interaction with IRE1B_LD_ (**Figure 4B**). A portion of the Strep protein alone present in the extract did not bind to HisPDI9 and was also detected in the flow-through (**Supplementary Figure S3**). Strep was not detected in the eluted fractions when using extracts from single vector controls of the Strep peptide on the column, suggesting binding was specific to HisPDI9 (**Supplementary Figure S3**). These results confirm that PDI9 interacted with the ER lumenal domains of IRE1A and IRE1B.

**Figure 5:**
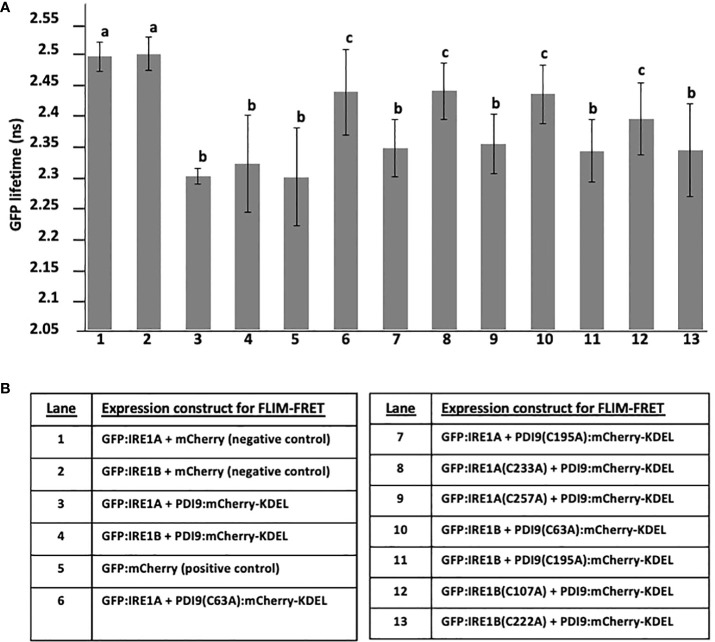
Summary of GFP lifetime values from the FLIM-FRET analysis of interactions between GFP : IRE1 and PDI9:mCherry-KDEL WT and mutants coexpressed in *Arabidopsis* protoplasts. The GFP lifetime values are presented in each lane on the *x*-axis in **(A)** for the expressed proteins listed by lane in **(B)**. In addition to WT PDI9 and IRE1A and IRE1B, the following mutant versions were analyzed: PDI9(C195A), PDI9(C63A), IRE1A(C233A), and IRE1B(C107A). The negative controls (lanes 1 and 2) consisted of the mCherry alone coexpressed with GFP : IRE1A or GFP : IRE1B. The statistical differences (*p*< 0.001, a–c) from one-way ANOVA and Tukey’s HSD *post-hoc* tests were calculated between samples coexpressing GFP : IRE1 and PDI9:mCherry-KDEL (including the respective mutants) with baseline GFP values in single- and dual-expressed samples with unfused mCherry alone. Error bars are shown as the standard deviation from two independent experiments of 30 to 50 cells per sample. Different lower case letters indicate statistical significance between groups.

Given that PDI9 is a redox-active protein folding enzyme, we next sought to investigate the role of the active site cysteine residues of PDI9 in their interaction with IRE1 in *Arabidopsis*. To accomplish this goal, site-specific mutagenesis was used to modify these key cysteine residues in PDI9 and IRE1 to the redox-inactive residue, alanine. For example, the second cysteine (Cys-63) in the first thioredoxin active motif of PDI9(CGHC) was mutated to alanine (CGHA, designated C63A). In the second thioredoxin active site motif of PDI9, the second cysteine (Cys-195) was also mutated to alanine (CGHC to CGHA, designated C195A). The single mutant versions of PDI9 were re-tested for potential interaction using *in vitro* co-I.P. firstly (**Figures 3**, **4**) and then *in vivo* FLIM-FRET analysis secondly (**Figure 5**). Interestingly, the C63A mutation in the first redox-active site of PDI nearly abolished interaction with IRE1A *in vitro*, as observed by immunoblot analysis from *in vitro* co-I.P. of HisPDI9(C63A) with StrepIRE1A_LD_ (**Figure 3B**). However, for the second C195A mutation of PDI9, the binding between HisPDI9(C195A) and StrepIRE1A_LD_ remained robust. The StrepIRE1A_LD_ was detected in the coeluted fractions with HisPDI9(C195A) at an almost similar level with respect to that observed when incubated with WT HisPDI9 (**Figure 3B**).

The converse experiment was next conducted, in which the key cysteines of IRE1A were mutated (C233A and C257A), and these mutant proteins tested for co-I.P. with the WT PDI9 of the HisPDI9 fusion. The StrepIRE1A_LD_(C233A) mutant exhibited diminished binding with HisPDI9 *in vitro*. Although the StrepIRE1ALD(C257A) mutant also exhibited diminished binding relative to WT StrepIRE1A_LD_, we observed slightly more binding of the IRE1A(C257A) to HisPDI9 compared to the IRE1A(C233A) mutant (**Figure 3B**).

Although a similar alteration in the binding pattern was also observed between the PDI9(C63A) mutant and IRE1B both *in vitro* and *in vivo*, the effect of the mutation was weaker relative to that with IRE1A (**Figures 4A, B**). In addition, whereas the C107A mutation in IRE1B reduced its interaction with HisPDI9, the IRE1B(C222A) mutation had only a minor effect on the interaction with HisPDI9 (**Figures 4A, B**).

To complement the co-I.P. approach to defining protein–protein interactions, the same protein mutations were subjected to FLIM-FRET analysis via transient expression in mesophyll protoplasts. The WT PDI9 sequence and two PDI9 mutations, C195A and C63A, were fused to mCherry-KDEL and tested to interact with the WT IRE1A sequence fused to GFP and the single C233A and C257A mutations of IRE1A, also fused to GFP (**Figure 5**). Likewise, the PDI9:mCherry-KDEL was tested to interact with the WT IRE1B fused to GFP and single mutant versions of IRE1B, C107A, and C222A fused to GFP (**Figure 5**). The two negative controls consisted of GFP : IRE1A and GFP : IRE1B, each coexpressed with mCherry alone. The positive control consisted of the direct fusion, GFP:mCherry.

The GFP lifetimes (**Figure 5**) for GFP : IRE1A and GFP : IRE1B coexpressed with mCherry alone were 2.49 ns and were comparable to GFP alone (**Figure 2**), indicating no GFP quenching in these negative controls. Significant quenching of donor GFP fluorescence was observed as 7.6% decreased lifetimes, 2.29 ns and 2.31 ns (**Figure 5**) when PDI9:Cherry-KDEL was coexpressed with either GFP : IRE1A or GFP : IRE1B, respectively. These quenching values were similar to the lower GFP fluorescence lifetime from the positive control GFP:mCherry (**Figure 5**, lane 5). The degree of quenching of the GFP donor fluorescence lifetime (2.42 ns) was decreased by half in protoplast cells coexpressing the mutant PDI9(C63A):mCherry-KDEL with GFP : IRE1A and PDI9(C63A):mCherry-KDEL with GFP : IRE1B (**Figure 5**, lanes 6 and 10). However, the lifetime values did not return to the same levels as the GFP : IRE1A control or when this control was dually expressed with the mCherry (no PDI9) control (**Figure 5**, lanes 1 and 2). Therefore, the C63A mutation in PDI9 decreased its affinities for IRE1A and IRE1B to intermediate levels. The C195A mutation in PDI9 (**Figure 5**, lanes 7 and 11) did not statistically alleviate the GFP lifetime quenching in the coexpressed pairs: PDI9(C195A):mCherry-KDEL with GFP : IRE1A; and PDI9(C195A):mCherry-KDEL with GFP : IRE1B.

We next examined the effects of single cysteine mutations in IRE1A (**Figure 5**, lanes 8 and 9) and IRE1B (**Figure 5**, lanes 12 and 13) on their ability to affect quenching of GFP lifetime fluorescence when paired with WT PDI9:mCherry-KDEL. The mutation C233A in IRE1A in the GFP : IRE1A(C233A) fusion when paired with PDI9:mCherry-KDEL pair (lane 8) significantly reduced the GFP fluorescence quenching. However, the C257A mutation in the fusion GFP : IRE1A(C257A) when paired with PDI9:mCherry-KDEL, did not statistically affect GFP fluorescence quenching. Similarly, the mutation C107A in IRE1B in the GFP : IRE1B(C107A) fusion, when paired with PDI9:mCherry-KDEL pair (lane 12), significantly reduced the GFP fluorescence quenching. Yet, the C222A mutation in the fusion GFP : IRE1B(C222A), when paired with PDI9:mCherry-KDEL, did not statistically affect GFP fluorescence quenching.

In summary, the FLIM-FRET results (**Figure 5**) indicate the C63A mutation in PDI9 was more effective than the C195A mutation in disrupting the quenching of GFP fluorescence. Likewise, the C233A mutation in IRE1A was more effective than the C257A mutation in affecting GFP lifetime fluorescence in the fusion pairing. However, for IRE1B, the C107A mutation was more effective than the C222A mutation.

### 3.3 PDI9 modulates UPR-dependent bZIP60 mRNA splicing

**Figure 6:**
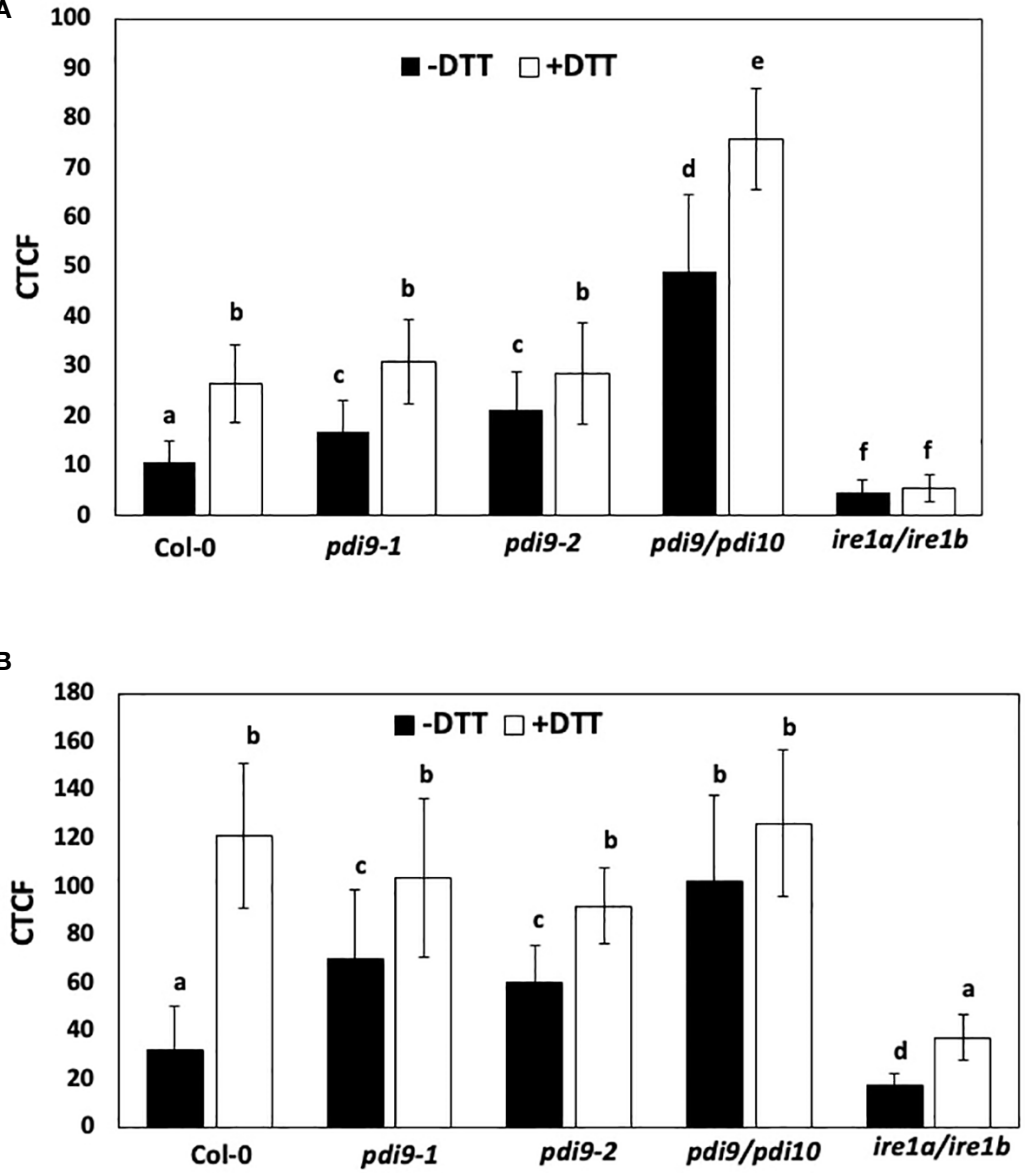
The use of two different reporter systems to measure the effects of DTT and the *pdi9* and *ire1* mutants on the expression of the UPR. **(A)** The first reporter measured the splicing of the *bZIP60* intron from the GFP mRNA in *Arabidopsis* protoplasts transiently expressing the 35S::*bZIP60* intron:GFP reporter construct (Carrillo and Christopher, 2022) under normal (−DTT) and ER stress (+DTT) conditions in the following genotypes: WT (Col-0), and the *pdi9-1*, *pdi9-2*, *pdi9–pdi10*, and *ire1a–ire1b* mutants. Protoplasts were cotransfected with the mCherry control to assess transfection efficiencies between cells. **(B)** The second reporter construct measured the expression of the UPR-responsive PDI9-promoter:mCherry in *Arabidopsis* protoplasts under normal (−DTT) and ER stress (+DTT) conditions in the following genotypes: WT (Col-0), and the *pdi9-1*, *pdi9-2*, *pdi9–pdi10*, and *ire1a–ire1b* mutants. Protoplasts cotransfected with GFP alone served as a control to assess transfection efficiencies between cells. Fluorescences of GFP and mCherry were observed by scanning confocal microscopy, and CTCF values were calculated using ImageJ for **(A**, **B)**. Statistical differences were analyzed from one-way ANOVA (*p*< 0.001) and Tukey’s HSD *post-hoc* tests between the samples. Error bars are shown as standard deviation from two independent experiments of 30 to 50 cells per sample. Representative protoplasts are shown in **Supplementary Figures S5**-**S8**. Different lower case letters indicate statistical significance between groups.

The intriguing evidence for a strong interaction of PDI9 with IRE1A and IRE1B suggests a potential role for PDI9 in the UPR signaling pathway. To test this hypothesis further, we examined the impact of the loss of the PDI9 gene family on UPR signaling by using a novel biosensor-reporter system that monitors UPR in living plant cells (Carrillo and Christopher, 2022). The intron from the *bZIP60* locus is spliced out in a UPR-dependent fashion. This intron was incorporated into the 5′-end of the GFP gene, creating the 35S::*bZIP60* intron:GFP construct. The *bZIP60* intron disrupts GFP mRNA from being translated under non-UPR conditions. Upon activation of UPR, the IRE1 kinase/ribonuclease splices the intron from the GFP RNA, permitting translation, resulting in GFP fluorescence. In **Figure 6A**, the effects of the PDI9 gene family on the UPR were studied via monitoring *bZIP60* intron splicing from the modified GFP in protoplasts of WT and the previously well-characterized single *pdi9-1*, *pdi9-2*, and double *pdi9–pdi10* mutant backgrounds (Feldeverd et al., 2020). The *ire1a–ire1b* double mutant was included as a control to ensure the measurement of GFP fluorescence and UPR-dependent bZIP60 intron splicing via an IRE-based signal transduction mechanism (**Figure 6A**).

CTCF levels of GFP were quantified in representative protoplasts from WT and mutant genotypes transfected with the 35S::*bZIP60* intron:GFP construct and treated with and without DTT (**Figure 6A**). Relative to WT protoplasts in the absence of DTT, significantly greater CTCF values were observed in protoplasts from the *pdi9-1*, *pdi9-2*, and *pdi9–pdi10* genotypes. Cells were chemically treated with the ER stress inducer, DTT, under short-term (3 h) conditions to avoid secondary effects of DTT (Carrillo and Christopher, 2022). In cells treated with DTT, all genotypes exhibited significantly greater CTCF values relative to nonstressed conditions. Most notably, relative to WT, the *pdi9–pdi10* double mutant exhibited nearly fivefold greater GFP fluorescence without DTT treatment and nearly threefold greater GFP fluorescence with DTT treatment (**Figure 6A**). CTCF levels were reduced to< 10% in the *ire1a–ire1b* double mutant (genotype confirmed in **Supplementary Figure S10**), indicating that GFP fluorescence and bZIP60 intron splicing were via the IRE-based signaling pathway (**Figure 6A**). There was no significant difference in transfection efficiency between the protoplasts expressing the 35S::*bZIP60* intron:GFP reporter used in this assay in either genotypes or treatments, as indicated by the cotransfected mCherry and GFP alone controls (**Figure 6A**). Representative protoplast cells expressing the 35S::*bZIP60* intron:GFP construct in the aforementioned genotypes and treatments are shown in **Supplementary Figures S5**, **S6**. These results indicate that the loss of PDI9 increases the activity of the UPR pathway and suggest that PDI9 attenuates the splicing of *bZIP60* mRNA through IRE1 RNase activity under ER stress.

### 3.4 The PDI9 promoter is induced during ER stress and in the *pdi9* mutants via the IRE1-mediated UPR pathway

A second test for the role of PDI9 in UPR signaling involved the use of the PDI9-promoter:mCherry reporter. The *PDI9* gene was previously shown to be transcriptionally induced by UPR via the bZIP60 transcription factor (Lu and Christopher, 2008). Therefore, the PDI9 promoter is a useful tool for measuring UPR signaling in WT, *pdi9-1*, *pdi9-2*, and *pdi9–pdi10* mutant backgrounds and would complement the aforementioned data (**Figure 6A**). This PDI9 promoter was incorporated upstream of the mCherry reporter gene, creating the PDI9-promoter:mCherry reporter construct. To further investigate the modulatory role of PDI9 within the UPR, this construct was transiently expressed in protoplasts from WT, *pdi9-1*, *pdi9-2*, *pdi9–pdi10*, and *ire1a–ire1b* genotypes with and without DTT treatments (**Figure 6B**). The mCherry expression was an indication of PDI9 promoter activity, and CTCF intensities were quantified per protoplast.

Relative to WT, the *pdi9-1*, *pdi9-2*, and *pdi9–pdi10* mutants exhibited significantly greater CTCF intensities in the absence of DTT (**Figure 6B**). The CTCF values were nearly threefold greater in the *pdi9–pdi10* genotype without DTT. DTT treatment markedly induced mCherry fluorescence in all genotypes relative to the untreated protoplasts. However, no significant difference was observed in CTCF values between WT and either *pdi9-1*, *pdi9-2*, or *pdi9–pdi10* mutants within the DTT treatments (**Figure 6B**). Therefore, the activation of the PDI9 promoter was upregulated under DTT-induced ER stress in all samples and also in the *pdi9-1*, *pdi9-2*, and *pdi9–pdi10* mutants under normal, noninduced conditions. The activation of the PDI9 promoter was severely reduced in both treatments in the *ire1a–ire1b* mutant. We observed no difference in fluorescence of the GFP transfection control when cotransfected with the PDI9-promoter:mCherry reporter in all genotypes under normal and ER-stress-induced conditions (**Figure 6B**). Representative protoplast cells expressing the PDI9-promoter:mCherry construct in the aforementioned genotypes and treatments are shown in **Supplementary Figures S7**, **S8**. Thus, transfection efficiencies remained relatively constant between genotypes and treatments. These results further validate that the PDI9 promoter is induced under ER stress via IRE1-mediated signaling. Furthermore, these results are relatively consistent with the results in **Figure 6A**, in which the loss of the function of the PDI9 gene family leads to an increase in UPR-regulated gene expression mediated by IRE1.

As additional internal controls for the *in vivo* reporter assays done in protoplasts, an analysis of the effects of the loss of PDI9 on UPR-regulated gene expression was also measured in protoplasts by qPCR (**Supplementary Figure S4**). Relative levels of RNAs from the UPR marker genes *BiP2*, *PDI11*, and *bZIP60* (*bZIP60t* and *bZIP60s* transcript variants) were measured in the protoplasts (treated with and without DTT) from WT (Col-0), *pdi9*, and *pdi9–pdi10* mutant backgrounds that were treated with DTT prior to qPCR analyses. All RNAs were higher in WT protoplasts exposed to the ER-stress inducer DTT, relative to untreated cells. However, relative to WT, the *BiP2*, *PDI11*, and *bZIP60* mRNAs were significantly elevated in the *pdi9* and *pdi9–pdi10* mutants under both normal (untreated) and ER stress-induced (DTT-treated) conditions (**Supplementary Figure S4**). The induction of RNA levels was greatest in the *pdi9–pdi10* double mutant. The removal of PDI9 and PDI10 leads to a marked increase in the levels of several key UPR gene transcripts. These qPCR results are directly consistent with the results from the two reporter assays, which are the 35S::*bZIP60* intron:GFP (**Figure 6A**) and PDI9-promoter:mCherry (**Figure 6B**) reporters. Taken together, these results indicate that PDI9 and PDI10 are negatively impacting the UPR pathway.

## 4 Discussion

### 4.1 PDI9 colocalizes and interacts with IRE1 in the ER lumen

We have previously determined that the nonclassical PDI-M subfamily member, PDI9, exhibited strong protein folding activity by disulfide bond-mediated folding of alkaline phosphatase *in vivo*, and it complemented the *dsbA* protein folding mutation in *E. coli* (Yuen et al., 2013). The PDI9-based protein folding activity is associated with pollen exine formation and protecting pollen development from heat stress (Feldeverd et al., 2020). In this study, we provided multiple lines of evidence demonstrating a new role for PDI9 in which it interacts in the ER with the UPR stress sensor, IRE1, in *Arabidopsis* and *E. coli* expression systems, which were simultaneously coexpressing PDI9 with either IRE1A or IRE1B homologs. These data are further supported by previous evidence of IRE1B localization to the ER in *Arabidopsis* protoplasts (Bao et al., 2018). The primary evidence for the physical interaction of PDI9 with full-length IRE1 was demonstrated *in vivo* in leaf mesophyll protoplasts via the stringent assay FLIM-FRET. FRET only occurs when the donor (GFP : IRE1) and acceptor (PDI9:mCherry-KDEL) proteins are in close physical proximity (< 10 nm) and in the proper polar orientation to permit energy transfer (Bajar et al., 2016; Kaufmann et al., 2020). FRET from the donor GFP : IRE1 to the acceptor PDI9:mCherry-KDEL was observed as a PDI9-mCherry-dependent quenching of GFP fluorescence. This decrease in the fluorescence lifetime of the GFP donor (**Figures 2**, **5**) is indicated by an increase in FRET efficiency. The FRET efficiencies of GFP : IRE1 with PDI9:mCherry-KDEL (**Figures 2**, **5**) ranged from 5.97% to 6.82% and were slightly less than the FRET efficiencies of 7.4% to 7.8% obtained with the positive control, GFP:mCherry direct translational fusion.

Further evidence supporting the interaction between PDI9 and IRE1 was obtained via co-I.P. of the proteins coexpressed in mesophyll protoplasts and in *E. coli.* The full-length versions of IRE1A and IRE1B were used in the plant protoplast expression system, whereas the lumenal domains of IRE1A and IRE1B were successfully expressed for co-I.P. in *E. coli*. The soluble ER lumenal domain of IRE1 is the region expected to be available to interact with PDI9, which also resides in the ER lumen (Feldeverd et al., 2020). In each expression system, the IRE1A and IRE1B proteins were detected in the co-I.P. fractions with PDI9 after stringent washes.

It is worth noting that we observed minor differences in the interaction patterns between IRE1A and/or IRE1B with PDI9. IRE1B displayed a consistently weaker interaction with PDI9 relative to IRE1A (**Figures 2**–**5**). It is hypothesized that functional and structural differences in IRE1B contribute to this difference in binding affinity with PDI9 relative to IRE1A. *Arabidopsis* IRE1A and IRE1B have functional redundancy, yet differences such as in expression patterns (Koizumi et al., 2001) and their roles in autophagy (Bao et al., 2018), pathogen response (Verchot and Pajerowska-Mukhtar, 2021), and gametogenesis (Pu et al., 2019) suggest specialization. The lumenal domains of the IRE1 homologs are less conserved among plant species relative to the cytosolic kinase/RNase domain. In *Arabidopsis*, IRE1A and IRE1B share 57% identity in the cytosolic domain and only 19% in the ER lumenal domain (Li and Howell, 2021). Therefore, PDI9 may preferentially bind to IRE1A in *Arabidopsis* as a result of structural or biochemical properties unique to the isoform. Questions remain as to what functional differences exist between IRE1A and IRE1B in the ER stress response, as well as what role PDI9 has with respect to these specializations.

### 4.2 PDI9 interacts with IRE1 primarily via C63 of the first thioredoxin domain of PDI9

Site-specific mutagenesis was used to individually disrupt single cysteines in each of the two thioredoxin catalytic domains of PDI9 and two critical cysteines each in IRE1A and IRE1B. The resulting co-I.P. and FLIM-FRET results with the mutated versions of PDI9 indicated that its interaction between IRE1A and IRE1B is largely dependent on the first thioredoxin domain’s active site (PDI9 C63), such that the C63A mutation abolished the interaction (**Figures 3**–**5**). Mutation of IRE1A (C233) and IRE1B (C107) sharply decreased interaction with WT PDI9. Therefore, the C63 may form a disulfide bond with IRE1 through a paired cysteine residue (IRE1A C233 and IRE1B C107) in the lumenal/sensor domain. In contrast, the C257A and C222A mutations in IRE1A and IRE1B, respectively, only slightly diminished interaction with PDI9 as opposed to the C233A and C107A mutations. Although PDI can engage in promiscuous disulfide introduction with various target cysteines (Yuen et al., 2013; Feldeverd et al., 2020), its isomerization and proofreading functions can rearrange disulfide bonds to the final most stable cysteine partners for oxidative binding, folding, and/or transfer of electrons in redox-based regulation of enzymes (Hatahet and Ruddock, 2009; Sato et al., 2013). We suggest that the cysteine positions in the lumenal domain of IRE1 may play a cooperative but nonequivalent role in binding to PDI9 for the proper structural conformation to enable a stable interaction.

Similarly, point mutations in the active sites of the mammalian ortholog, PDIA6, reveal that the first N-proximal CXXC active site motif exhibits greater catalytic activity with respect to the C-terminal active site (Lyles and Gilbert, 1994). The mammalian ortholog PDIA6 was shown to interact with IRE1 through the critical cysteine residue Cys148 (Liu et al., 2003; Eletto et al., 2014) and form a disulfide bond with PDIA6, and this interaction was dependent on the phosphorylation-based dimerization and activation of IRE1 (Eletto et al., 2014). The influence of PDI9 on the phosphorylation-activation status of IRE1 in plants has not been determined. However, the data with the PDI9 gene knockout mutants described below supports the hypothesis that PDI9 attenuates IRE1 activity.

### 4.3 The loss of PDI9 has a stimulatory effect on the IRE1-mediated branch of the UPR signaling pathway

**Figure 7:**
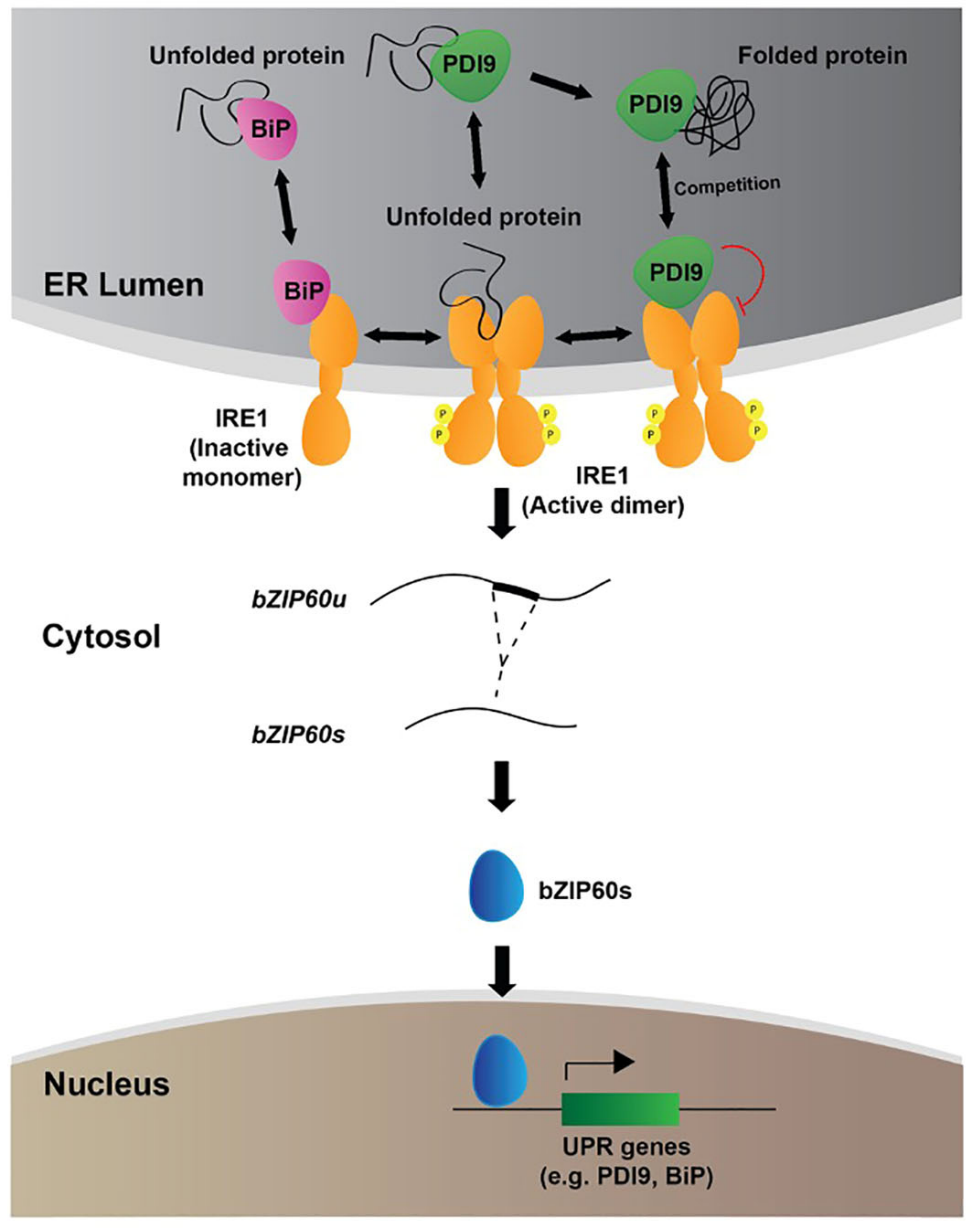
Proposed model for the role of PDI9 in the UPR in *Arabidopsis*. The following three subcellular zones of relevancy to the UPR are shown: the ER; the cytosol; and the nucleus. The locations and roles of BiP (pink), IRE1 (orange), PDI9 (green), and bZIP60 (blue) are labeled. IRE1 spans the ER membrane with lumenal and cytosolic domains and exists in monomeric and active dimeric forms. The small circled “P” denotes phosphorylation of dimeric IRE1 on the cytosolic side. The folded and misfolded proteins are denoted with black entwined and semi-entwined lines, respectively. The red curved line indicates repression. Double-arrowheaded lines show reactions that flow in both directions.

Three distinct experimental approaches were used to indicate that the loss of PDI9 resulted in the upregulation of UPR gene expression. Each approach targets a different step in the UPR pathway. First, the absence of PDI9 increased the UPR-dependent splicing of the *bZIP60* intron from the GFP mRNA when the 35S::*bZIP60* intron:GFP reporter was expressed in protoplasts. This led to significantly higher levels of GFP fluorescence detected in the two single *pdi9* mutants and the double *pdi9–pdi10* mutant relative to WT under normal conditions (**Figure 6**). Moreover, with respect to WT, a near threefold elevation of *bZIP60* mRNA intron splicing was observed in *pdi9–pdi10* cells under DTT-induced ER stress (**Figure 6**). This splicing is one of the first steps in IRE1 kinase/RNase output at the start of the UPR signal transduction pathway. Secondly, the activation of the PDI9 promoter, which is a downstream target of the IRE1-induced *bZIP60* transcription factor in UPR signaling (Lu and Christopher, 2008), was also highly upregulated in the single and double mutants in the absence of ER stress, and in all genotypes under ER stress (**Figure 7**). Thirdly, in leaf mesophyll protoplasts, mRNA levels significantly increased for UPR-induced chaperones *BiP2* and *PDI11* plus *bZIP60* total and spliced RNAs in the single *pdi9* and double *pdi9–pdi10* mutants relative to WT with and without DTT treatment (**Supplementary Figure S4**). Thus, taken together, we conclude that PDI9 plays an important role in the UPR, and its loss leads to a significant stimulation of the UPR pathway and downstream UPR-regulated gene expression.

### 4.4 UPR hyperactivation: compensation and attenuation mechanisms in the ER control the output of IRE1 signaling and the UPR

This investigation raises a major question about the mechanism by which the loss of PDI9 leads to the hyperactivity of the UPR. We propose the following dual roles for PDI9: (a) the compensation mechanism; and (b) the attenuation mechanism. The first, PDI9, has been well-documented to catalyze oxidative disulfide bond-mediated protein folding in the ER (Yuen et al., 2013; Feldeverd et al., 2020). Therefore, the loss of PDI9 in the *pdi9* knockout mutants results in a deficiency of PDI9-mediated protein folding capacity, leading to more unfolded proteins in the ER. This increased demand for protein folding could be satisfied by a general stimulation of UPR and the accumulation of downstream protein folding chaperones. The resulting stimulation of UPR partially compensates for the PDI9 deficiency. In the second mechanism, we postulate that PDI9 interacts with the lumenal domain of IRE1 in the ER to attenuate the IRE1-mediated UPR pathway. The loss of PDI9, therefore, derepresses IRE1, leading to stimulation of UPR and its downstream components.

The model in **Figure 7** depicts in further detail the proposed dual mechanisms by which PDI9 fits into the current thinking of UPR. Under unstressed conditions, the Hsp70 cognate, BiP, associates with the lumenal face of monomeric IRE1, preventing its activation and phosphorylation-based dimerization (Kopp et al., 2018). A low level of basal splicing of *bZIP60* mRNA occurs through the IRE1 dual kinase/RNase activity (Eletto et al., 2014). In response to ER stress, however, unfolded and misfolded proteins accumulate and bind to BiP (Bravo et al., 2013; Vitale et al., 2019). BiP then dissociates, liberating IRE1 to dimerize and activate the UPR (Adams et al., 2019). It is thought that the IRE1 lumenal domain can also bind to the exposed hydrophobic residues on unfolded/misfolded polypeptides, triggering dimerization and subsequence activation of the cytosolic kinase and RNase domains (Li and Howell, 2021). Activated IRE1 catalyzes the unconventional splicing of *bZIP60* mRNA in the cytosol, recognizing and cleaving two characteristic RNA stem-loop structures in a 23-nt intron. This *bZIP60* RNA intron maintains an encoded reading frame for a C-terminal transmembrane domain that normally localizes the protein to the ER membrane. However, splicing out the intron-encoding sequence produces a new translational reading frame for the *bZIP6*0 transcription factor that translocates to the nucleus to activate UPR target genes. Induction of such genes is mediated through the ER stress response element (ERSE) and UPR element (UPRE), *cis*-regulatory sequences located in their promoters (Iwata and Koizumi, 2012).

During this process, we propose two competing molecular functions of PDI9 that govern the regulatory system. One is involved in oxidative protein folding, and the other binds to dimeric IRE1 to attenuate IRE1 signaling. The stoichiometric balance of these PDI9 activities maintains proteostasis in the ER between stressed and unstressed conditions. When the PDI9 population is consumed by a high demand for protein folding, less PDI9 is available to interact with the IRE1 complex, thus activating UPR. The need for UPR and protein folding would be high in this scenario of ER stress. In turn, as ER stress and the demand for protein folding subside, less PDI9 is involved in protein folding. A free population of PDI9 is thus available that can associate with the IRE1 complex to attenuate it. The need for UPR would be diminished in this phase.

This layered and multiplex attenuation prevents excessive and continuous UPR signaling, which can be deleterious (Lin et al., 2007; Rubio et al., 2011; Tufo et al., 2014). For example, attenuation of yeast UPR is essential for survival and is mediated by inhibition of *IRE1* (Chawla et al., 2011). Research suggests that excessive UPR signaling can promote programmed cell death (PCD) in plants when ER stress remains constitutive (Manghwar and Li, 2022), highlighting the importance of high sensitivity and responsiveness with respect to both the activation and attenuation of this essential signaling pathway (Manghwar and Li, 2022). The model (Eletto et al., 2014) for the human homolog of *Arabidopsis* PDI9, PDIA6, denotes that it interacts with IRE1 to limit the duration of UPR activity and inhibit the response. However, unlike for plant PDI9 (Yuen et al., 2013), no protein folding activity has been detected for human PDIA6. Due to the importance of the UPR (Zhang et al., 2016) and the conserved primary structure of the PDIs between plants and other eukaryotes (Yuen et al., 2013), we suggest the basic regulatory mechanism of a PDI that represses IRE1 could be conserved among eukaryotes. This mechanism modulates the output of UPR and highlights the critical need for multiple forms of regulation of the ER stress response, including a safeguarding mechanism for attenuation to ensure cellular homeostasis is maintained.

Although the interaction of PDI9 with IRE1 is especially strong, it does not preclude that other PDIs in Arabidopsis could interact with IRE1A/B and influence IRE1A/B and UPR. It is conceivable that simultaneously knocking out other PDI genes could decrease protein folding activity and thereby increase the UPR. Further experimentation would be required to support the broader role of the entire PDI family, which would include interaction and gene knockout studies. However, in mammals, only the PDI9 homolog PDIA6 interacts with IRE1 (Eletto et al., 2014). In addition, we conducted quantitative yeast-two-hybrid screening of Arabidopsis PDI1 and PDI2 (Cho et al., 2011) and PDI5 (Ondzighi et al., 2008) and found no interaction with IRE1. The studies reported here provide a solid foundation for further experimentation to test the proposed model.

With the exception of BiP (Humbert et al., 2012; Li and Howell, 2021), much is unknown regarding the factors that regulate the UPR once activated in plants, particularly within the ER lumen. In mammals, proteins have been identified that function to attenuate the UPR from the cytosolic face of activated IRE1, including the Bcl-2 family protein, BI-1, and several phosphatases (Eletto et al., 2014). Zhu et al. (2019) recently showed that the anti-apoptotic-like proteins GAAP1 and GAAP3 interact with the cytoplasmic domain of IRE1A and IRE1B in *Arabidopsis* to negatively modulate the UPR and reduce the extent of ER stress-induced cell death. However, the UPR originates from inside the ER and responds to the dynamic secretory protein load in the lumen. This investigation sheds light on the PDI9-based mechanisms by which the unfolded protein signals in the ER are being communicated to ensure an appropriate UPR. In summary, this work highlights the importance of understanding how plants regulate the intricacies of the ER stress response to maintain and protect cellular homeostasis. We propose a model of the UPR pathway in plants in which PDI9 binds to IRE1 in the ER lumen to modulate the UPR pathway. This interaction takes place largely through the formation of a critical disulfide bond between the two protein partners and ultimately affects downstream splicing of the UPR-targeted transcription factor, *bZIP60*, as well as the expression of UPR-targeted genes such as BiP and PDI9 itself. PDI9 also functions as a critical foldase (Yuen et al., 2013) to assist with the oxidative folding of nascent or misfolded polypeptides within the ER involved in pollen exine formation (Feldeverd et al., 2020). The subgroup of PDI9 engaged in protein folding influences the amount of PDI9 available for the PDI9–IRE1 interaction identified here. The balance of such PDI9 activities, therefore, serves as an important signaling mechanism for the cell that regulates the protein folding load to maintain homeostasis and prevent excessive UPR signaling in an adaptive manner. The multiplex ability of the cell to modulate and control such a critical stress signaling pathway would promote the levels of UPR to be precisely proportional to the demand. These results highlight the need for better understanding the intricate mechanisms by which plants mitigate ER stress-inducing events such as heat stress and promote future agricultural advancements that may improve plant tolerance to stress.

## Data availability statement

The original contributions presented in the study are included in the article/**Supplementary Files**. Further inquiries can be directed to the corresponding author.

## Author contributions

RC: Data curation, Formal analysis, Investigation, Methodology, Validation, Visualization, Writing – original draft, Writing – review & editing. KI: Formal analysis, Funding acquisition, Investigation, Resources, Writing – review & editing. AA: Funding acquisition, Investigation, Resources, Writing – review & editing. GD: Investigation, Methodology, Writing – review & editing. DC: Conceptualization, Formal analysis, Funding acquisition, Project administration, Resources, Supervision, Validation, Visualization, Writing – original draft, Writing – review & editing.
