# Supplementary material for: Protein disulfide isomerase-9 interacts with the lumenal region of the transmembrane endoplasmic reticulum stress sensor kinase, IRE1, to modulate the unfolded protein response in Arabidopsis
Source: Front Plant Sci. 2024 May 16;15:1389658. doi: 10.3389/fpls.2024.1389658 (PMC11137178; doi:10.3389/fpls.2024.1389658)
Supplement: Supplementary file 1 [file DataSheet_1.pdf]

```
PDI9    6     LTLLTLLTICFGFFDLSSALYGSSSPVVQLTASNFKSKVLNSNGVVLVEFFAPWCGHCKA  65
PDIA6   1     MALLVLGLVSCTFFLAVNGLYSSSDDVIELTPSNFNREVIQSDSLWLVEFYAPWCGHCQR  60

PDI9    66    LTPTWEKVANILKGVATVAAIDADAHQSAAQDYGIKGFPTIKVFVPGK-APIDYQGARDA  124
PDIA6   61    LTPEWKKAATALKDVVKVGAVDADKHHSLGGQYGVQGFPTIKIFGSNKNRPEDYQGGRTG  120

PDI9    125   KSIANFAYKQIKGLLSDRLEGKSKPTGGGSKEKKSEPSAS---VELNASNFDDLVIESNE  181
PDIA6   121   EAIVDAALSALRQLVKDRLGGRSGGYSS-GKQGRSDSSSKKDVIELTDDSFDKNVLDSED  179

PDI9    182   LWIVEFFAPWCGHCKKLAPEWKRAAKNLQ----GKVKLGHVNCDVEQSIMSRFKVQGFPT  237
PDIA6   180   VWMVEFYAPWCGHCKNLEPEWAAAASEVKEQTKGKVKLAAVDATVNQVLASRYGIRGFPT  239

PDI9    238   ILVFGPDKSSPYPYEGARSASAIESFASELVESSAGPVEVTELTGPDVMEKKCGSAAICF  297
PDIA6   240   IKIFQKGES-PVDYDGGRTRSDIVSRALDLFSDNAPPPELLEIINEDIAKRTCEEHQLCV  298

PDI9    298   ISFLPDILDSKAEGRNKYLEMLLSVAEKFKKQPYSFMWVAAVTQMDLEKRVNVGGYGYPA  357
PDIA6   299   VAVLPHILDTGAAGRNSYLEVLLKLADKYKKKMWGWLWTEAGAQSELETALGIGGFGYPA  358

PDI9    358   MVAMNVKKGVYAPLKSAFELQHLLEFVKDAGTGGKGNVPMNGT--PEIVKTKEWDGKDGE  415
PDIA6   359   MAAINARKMKFALLKGSFSEQGINEFLRELSFGRGSTAPVGGGAFPTIVEREPWDGRDGE  418

PDI9    416   LIEEDEFSLDELMGGDDAVGSKDEL 440
PDIA6   419   LPVEDDIDLSDVELDDLG---KDEL 440
```

Supplemental Fig. S1: Arabidopsis PDI9 is 62% homologous to human PDIA6. Alignment of PDI9 and PDIA6 amino acid sequences. Identical and similar residues are presented in bold. The two thioredoxin “CGHC” motifs are underlined.

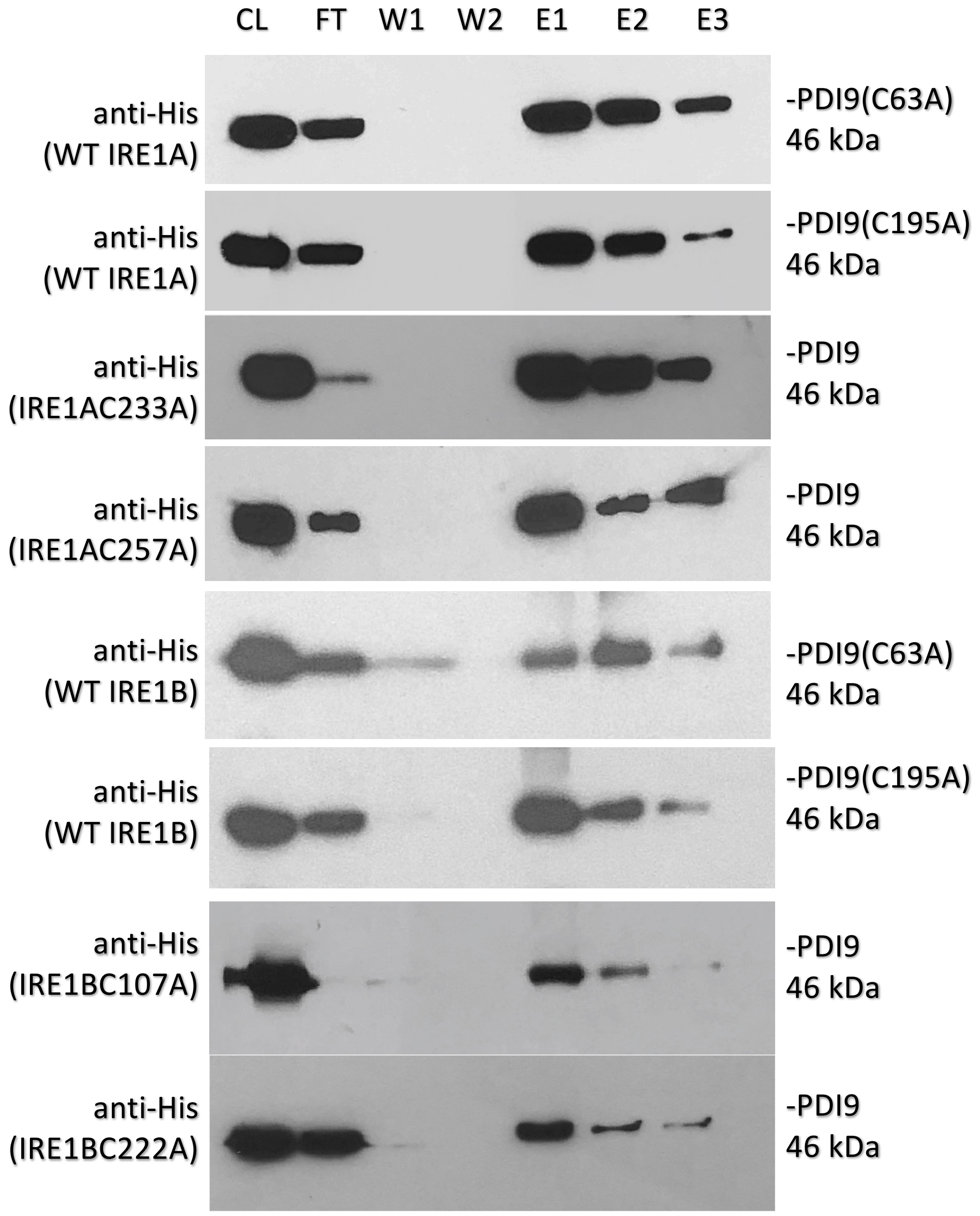

Supplemental Fig. S2: Anti-His Western blots for *in vitro* anti-His co-I.P. experiments testing interaction between His-tagged PDI9 and Strep-tagged $IRE1_{LD}$ variants. Affinity chromatography was done using HisPDI9 as bait, with the specific construct denoted on the right. The co-incubated and putative protein interactor (the $StrepIRE1_{LD}$ variants) are denoted on the left of each respective anti-His blot. The complimentary anti-Strep Western blots, including the location of each cysteine-to-alanine mutation indicated, are shown in Fig. 3 and Fig. 4. Abbreviations: Crude lysate (CL), Flow through (FT), Wash 1-2 (W1, W2), Elution 1-3 (E1, E2, E3).

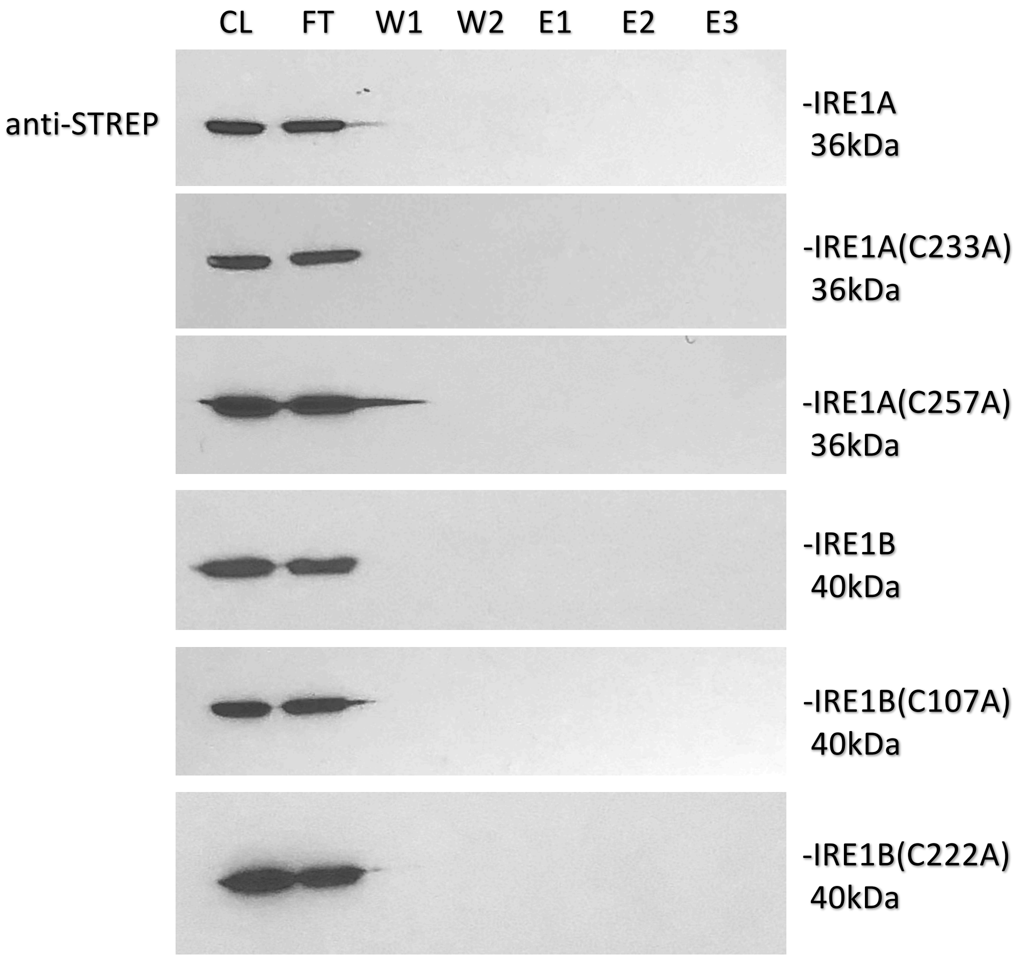

Supplemental Fig. S3: Anti-Strep Western blots of negative control StrepIRE1$_{LD}$ samples for *in vitro* anti-His co-I.P. experiments testing interaction between His-tagged PDI9 and Strep-tagged IRE1$_{LD}$ variants. The StrepIRE1$_{LD}$ constructs (denoted on the right of each blot) were expressed as a single protein and anti-His affinity chromatography was performed to show that non-specific binding of the respective prey protein in each experiment did not occur. The complimentary anti-His and anti-Strep Western blots, including the location of each cysteine-to-alanine mutation indicated, are shown in Fig. 3 and Fig. 4. Abbreviations: Crude lysate (CL), Flow through (FT), Wash 1-2 (W1, W2), Elution 1-3 (E1, E2, E3).

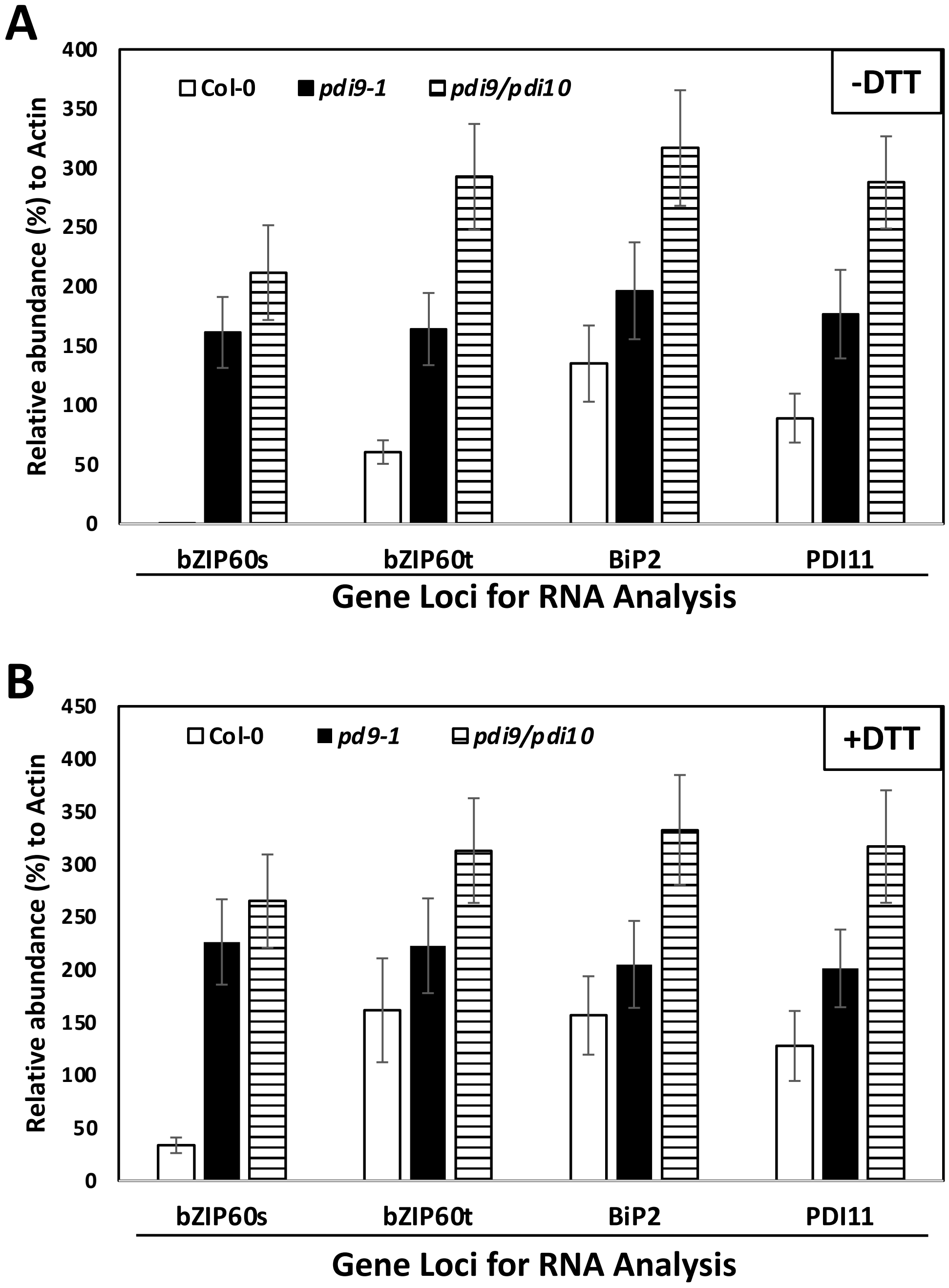

Supplemental Fig. S4: Traditional RT-PCR (A,B) analysis of UPR-responsive genes in *Arabidopsis* protoplasts from WT (Col-0), *pdi9-1*, *and pdi9-pdi10* genotypes under normal (-DTT, panel A) and ER stress (+DTT, panel B) conditions. Relative RNA abundances (%) are shown for the following UPR markers: spliced *bZIP60* mRNA (*bZIP60s*); total *bZIP60* RNA (*bZIP60t,* both spliced plus unspliced forms); binding protein, *BiP2;* and the protein folding chaperone, *PDI11.* The RNA levels are represented on an equal basis relative to the RNA levels for the internal control, *Actin2.*

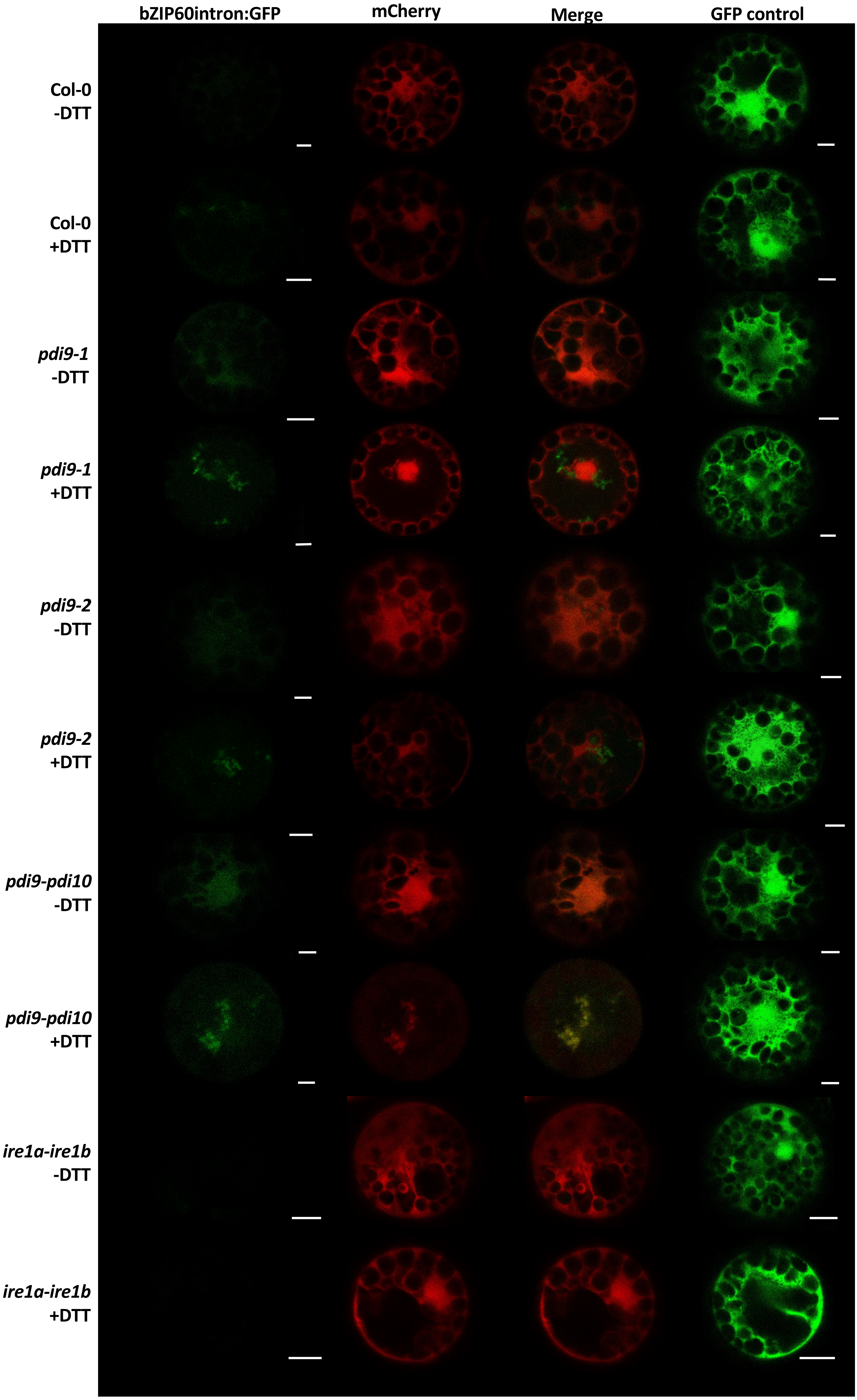

Supplemental Fig. S5: The effects of DTT and the *pdi9* and *ire1* mutants on the splicing of the *bZIP60* intron from the GFP mRNA in *Arabidopsis* protoplasts. Representative protoplast cells transiently expressing the 35S::*bZIP60* intron:GFP reporter construct (Carrillo and Christopher, 2022) under normal (-DTT) and ER stress (+DTT) conditions in the following genotypes: WT (Col-0), and the *pdi9-1*, *pdi9-2*, and *pdi9-pdi10, and ire1a-ire1b* mutants. Protoplasts were co-transfected with the mCherry control to assess transfection efficiencies between cells when untreated (-DTT) and treated with DTT (+DTT). The GFP, mCherry, and a merge of the two channels are shown. A representative cell from a single transfection with the GFP control vector is also illustrated showing cytoplasmic accumulation of GFP. Scale bars are indicated at 0-5 μ.

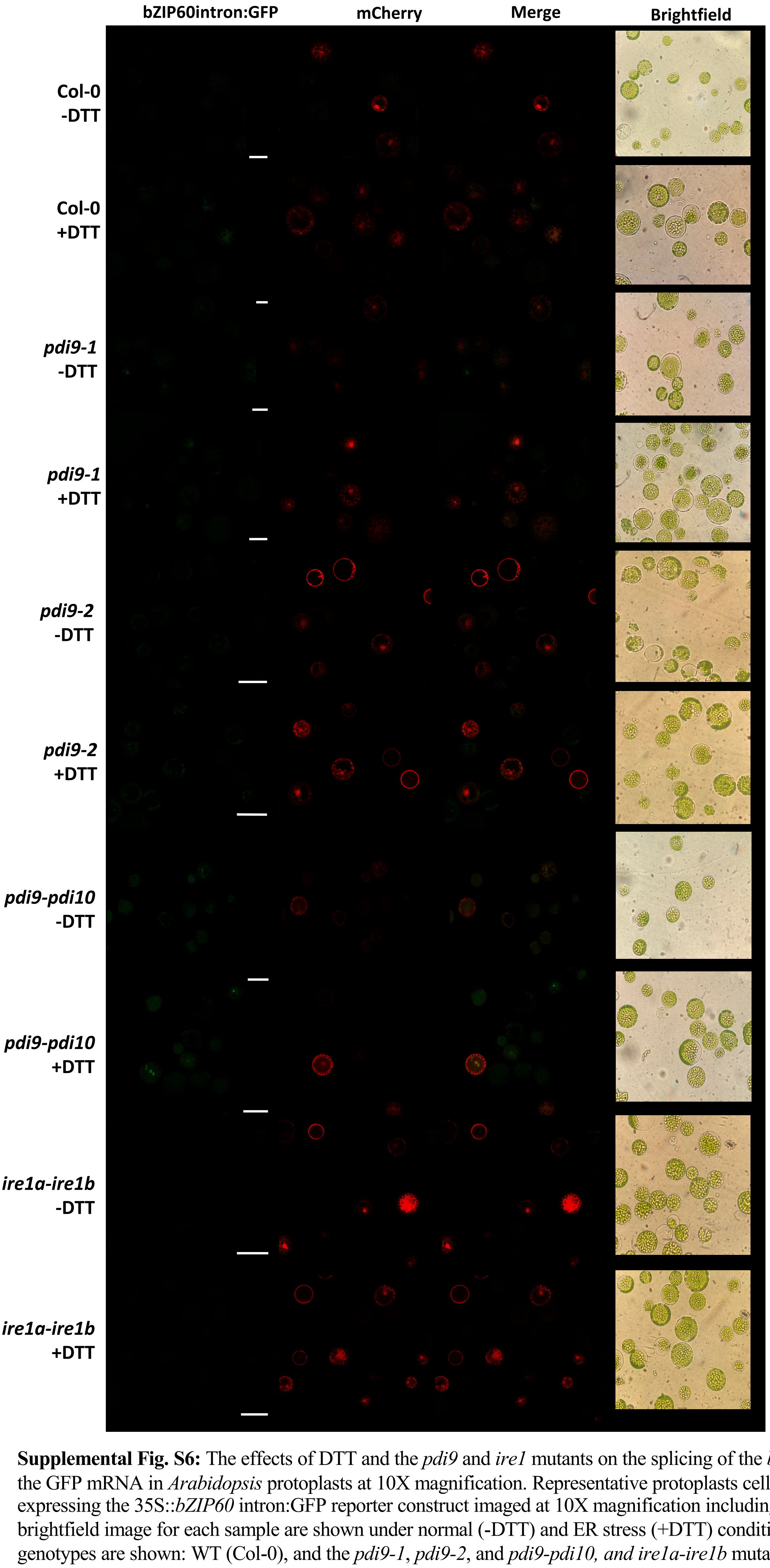

Supplemental Fig. S6: The effects of DTT and the *pdi9* and *ire1* mutants on the splicing of the *bZIP60* intron from the GFP mRNA in *Arabidopsis* protoplasts at 10X magnification. Representative protoplasts cells transiently expressing the 35S::*bZIP60* intron:GFP reporter construct imaged at 10X magnification including the respective brightfield image for each sample are shown under normal (-DTT) and ER stress (+DTT) conditions. The following genotypes are shown: WT (Col-0), and the *pdi9-1*, *pdi9-2*, and *pdi9-pdi10, and ire1a-ire1b* mutants. Protoplasts were co-transfected with the mCherry control to assess transfection efficiencies between cells when untreated (-DTT) and treated with DTT (+DTT). The GFP, mCherry, a merge of the two channels, and a brightfield image are shown. Scale bars are indicated at 0-5 μ.

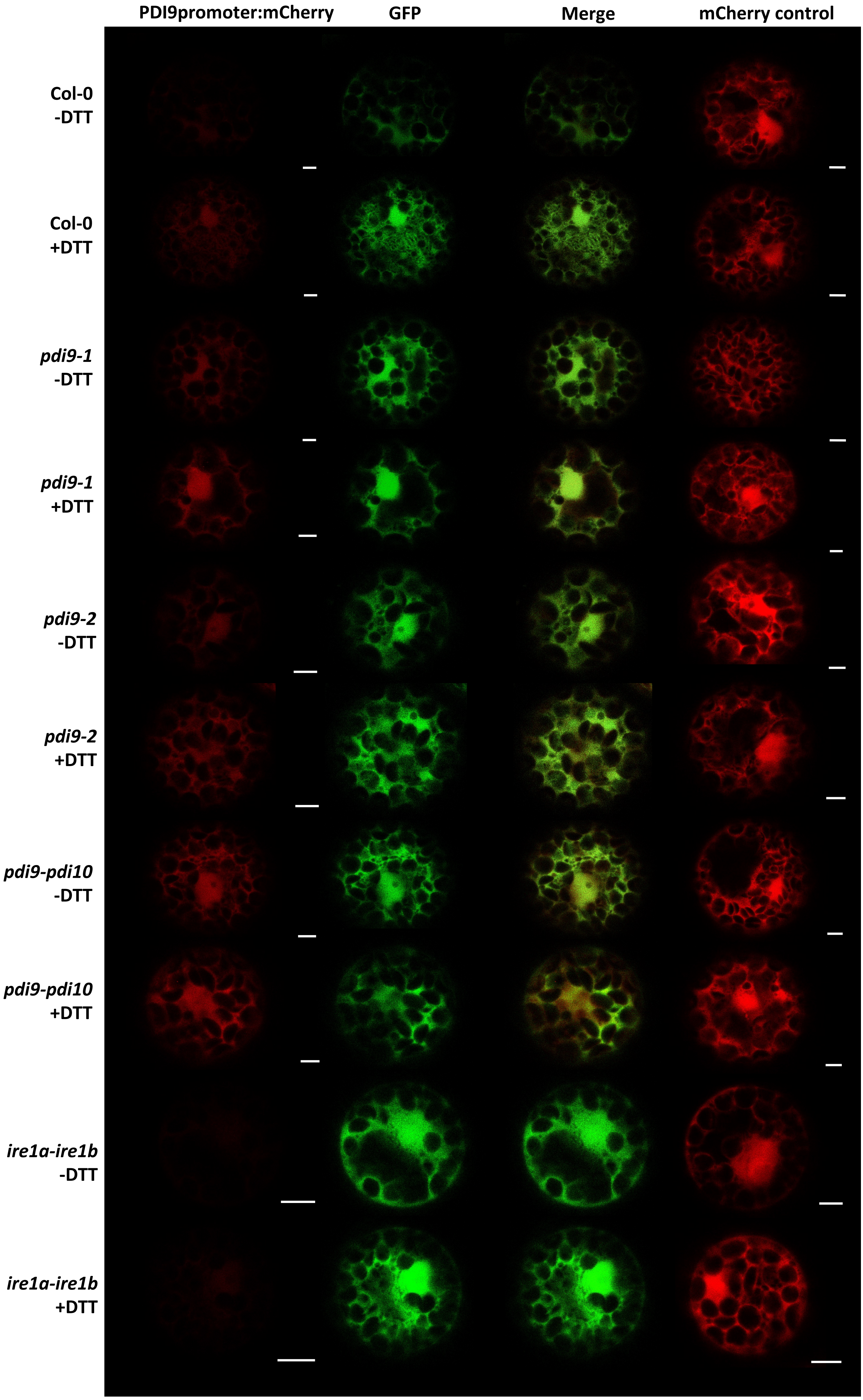

Supplemental Fig. S7: Analysis of the effects of DTT and *pdi9* mutants on the expression of the PDI9-promoter:mCherry reporter construct in *Arabidopsis* protoplasts. Representative protoplasts from the WT (Col-0), and the *pdi9-1*, *pdi9-2, pdi9-pdi10* and *ire1a-ire1b* mutants are shown expressing the PDI9-promoter:mCherry reporter construct under normal (-DTT) and ER stress (+DTT) conditions. Protoplasts co-transfected with GFP alone served as a control to assess transfection efficiencies between cells. The mCherry, GFP, and merge of the two channels are shown. A representative cell from a single-transfection with the mCherry control vector is also illustrated showing cytoplasmic accumulation of mCherry. Scale bars are indicated at 0-5 μm. Expression and fluorescence of mCherry was observed by scanning confocal microscopy

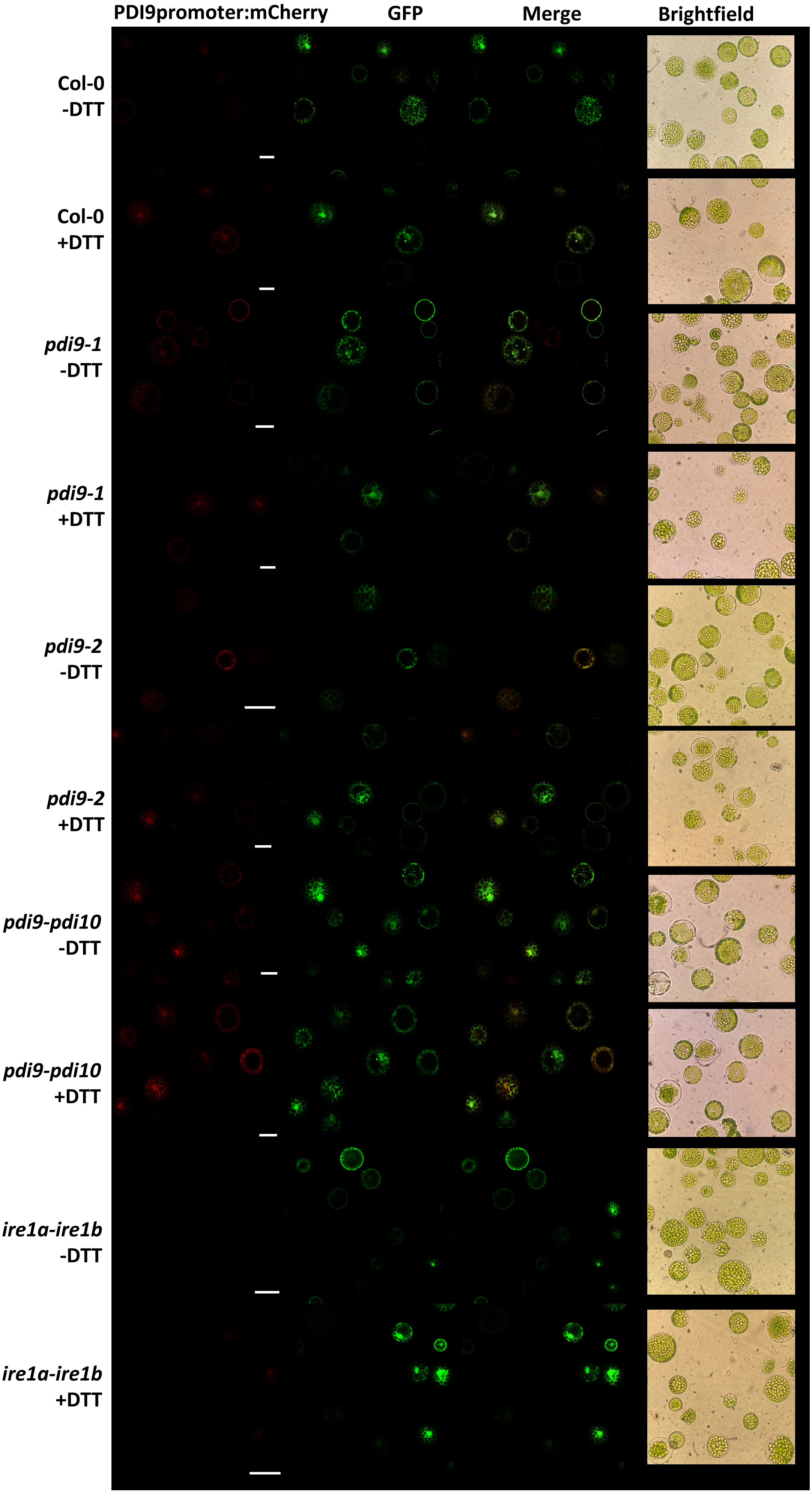

Supplemental Fig. S8: The effects of DTT and the *pdi9* and *ire1* mutants on the expression of the PDI9-promoter:mCherry reporter construct in *Arabidopsis* protoplasts at 10X magnification. Representative protoplasts cells transiently expressing the reporter construct imaged at 10X magnification including the respective brightfield image for each sample are shown under normal (-DTT) and ER stress (+DTT) conditions. The following genotypes are shown: WT (Col-0), and the *pdi9-1*, *pdi9-2*, and *pdi9-pdi10, and ire1a-ire1b* mutants. Protoplasts were co-transfected with the GFP control to assess transfection efficiencies between cells when untreated (-DTT) and treated with DTT (+DTT). The GFP, mCherry, a merge of the two channels, and a brightfield image are shown. Scale bars are indicated at 0-5 μ.

Primer	Sequence, 5' to 3'
KpnI-35S-F	CACGGTACCTTGAGACTTTTCAACAAAGGGTAATATC
XhoI-35S-R	CAACTCGAGGTGTTCTCTCCAAATGAAATGAAC
XhoI-IRE1A5'UTR-F	ATACTCGAGCCGGACCATGCCGCCGAGATGTCCTTTCC
ClaI-IRE1Asp-R	GAAATCGATGTCGGCAGCGCCGCCGCAAGGAG
ClaI-GFP-F	AACATCGATATGGTGAGCAAGGGCGAGGAGCTG
XmaI-GFP-R	ATTCCCGGGCTTGTACAGCTCGTCCATGCCGAGAG
XmaI-IRE1Amatpep-F	ACCCCCGGGGATGTGACGTATCCGATCGTTC
BamHI-IRE1A-R	ACAGGATCCTTAGATGATGTCGCATTTGAAGTACTTTCTG
BamHI-Nos-F	GCAGGATCCCGATCGTTCAAACATTTGGCAATAAAG
NotI-Nos-R	ATAGCGGCCGCCCGATCTAGTAACATAGATGACACC
XhoI-IRE1B5'UTR-F	CAGCTCGAGCTAAAGCGATGAGAGGATCTGCACTACTTGATTTG
ClaI-IRE1Bsp-R	GAAATCGATGCTGTGTGCTAATGGAGATACGAG
XmaI-IRE1Bmatpep-F	CCACCCGGGCACAGCTTCAAAGGATCTGAAATCTCC
NotI-IRE1B-R	CCAGCGGCCGCCTAGAATACAGTGGTCTTAGAGTACTTG
XhoI-mCherry-F	ATTCTCGAGATGGTGAGCAAGGGCGAGGAGG
BamHI-mCherry-R	CTTGGATCCTTACTTGTACAGCTCGTCCATG
NotI-Nos-F	ACCGCGGCCGCGATCGTTCAAACATTTGGCAATAAAG
SacI-Nos-R	CAAGAGCTCCCGATCTAGTAACATAGATGACACC
GFP(Spe)F	ATCACTAGTAAAAAAATGGTGAGCAAGGGCGAGGA
GFP(BamHIXmaI)R	AAAGGATCCCCCGGGCTTGTACAGCTCGTCCATGC
EcoRI-35S-F	GGCGAATTCTTGAGACTTTTCAACAAAGGGTAATATC
KpnI-35S-R	CCAGGTACCGTGTTCTCTCCAAATGAAATGAAC
KpnI-sp-PDI9-R	ACCGGTACCGAAAGAGAGAAAGGGGAAGAAGAAAAATGTATAAATC
BamHI-HAPDI9-R	CACGGATCCAGCGTAATCTGGAACATCGTATGGGTATCCATAAAGAGCACTGCTGAG ATC
BamHI-PDI9-F	ACAGGATCCATCTTCGTCACCTGTGGTTCAG
NotI-PDI9-R	TCTGCGGCCGCCTTGGATTCTTCACAACTCATC
KpnI-StrepIRE1A(LD)-F	ATCGGTACCTGGAGCCACCCGCAGTTCGAAAAGTCTCTTTATGGATCTTCGTCACCTG TGGATGTGACGTATCCGATCGTTC
PacI-IRE1A-R	CGCTTAATTAACTAAGTTTGAAGTTTAGGACCGAGGTC
KpnI-StrepIRE1B(LD)-F	CTCGGTACCTGGAGCCACCCGCAGTTCGAAAAGTCTCTTTATGGATCTTCGTCACCTG TGAGCTTCAAAGGATCTGAAATCTC
PacI-IRE1B-R	ACCTTAATTAACTATTTGCTAGCAAAGCCTGCCTG
KpnI-PDI9-Prom-F	GTTGGTACCGTAGTTTGGATAGTTGATGCAC
XhoI-PDI9-Prom-R	TATCTCGAGTTCTTCCCCTTTCTCTC
PDI9-TRX-A63-F	TGGTGTGGTCATGCGAAAGCTCTAACACCTACATGGGAG
PDI9-TRX-A63-R	AGCTTTCGCATGACCACACCATGGTGCAAAGA
PDI9-TRX-A195-F	CCTTGGTGTGGACATGCGAAAAAGCTTGCCCCGGAGTGGAAG
PDI9-TRX-A195-R	AAGCTTTTTCGCATGTCCACACCAAGGTGCAAAAAACTCAAC
IRE1A-A233-F	CTTCAGAGCTGCTTTACTAGCAGATCCTG
IRE1A-A233-R	CATAGCCAGAATTGAAAACAGGATCTGCTAGTAAAG
IRE1A-A257-F	CTGGAATTTATATGCCATTGTTAGCAGGATC
IRE1A-A257-R	CGAACGTCAATTTGTGATCCTGCTAACAATG
IRE1B-A107-F	GTGATGACTTTTACATGGATGCGGATAAAG
IRE1B-A107-R	GTAAAGACGCCAATCTTTATCCGCATCC
IRE1B-A222-F	ATACAAGCGATATCAAAGTTTGGAGATG
IRE1B-A222-R	TTGATATCGCTTGTATTTTAAAATCTTTTCTCTC
PDI9-F	ACAGGATCCATCTTCGTCACCTGTGGTTCAG
PDI9-R	TCTGCGGCCGCCTTGGATTCTTCACAACTCATC
bZIP60-F	GAAGGAGACGATGATGCTGTGGCT
bZIP60-R	GCAAATGAAGTTTACTCCCAGAAGCCAAAGCAGG
bZIP60s-R	AGCAGGGAACCCAACAGCAGACTC
BiP2-F	ACCCTCGAGATGGCTCGCTCGTTTGGAGCAAACAG
BiP2-R	CCAGACGTCCTAGAGCTCATCGTGAGACTCATC
Actin2-F	TCCTTGTACGCCAGTGGTCG
Actin2-R	CCGCTCTGCTGTTGTGGTGA
LBa1	TGGTTCACGTAGTGGGCCATCG
SALK_002316_ire1a-3_RP	GTCTGAAGCTTCTGGAACGTG
SAIL_LB2	GCTTCCTATTATATCTTCCCAAATTACCCATAC
SAIL_238_F07_RP	GAAGGAAAACGGACATCCTTC
SALK_002316_ire1a-3_LP	ACCTCATTGCATCAGACCAAC
IRE1B-Int2-Forward	TCTAGACGAGGCGTTGTATCTTC
IRE1B-Int4-Reverse	GAAGATGAACAGCTTCTGGAAGAC

Supplemental Fig. S9: The list of all primers used in this study.

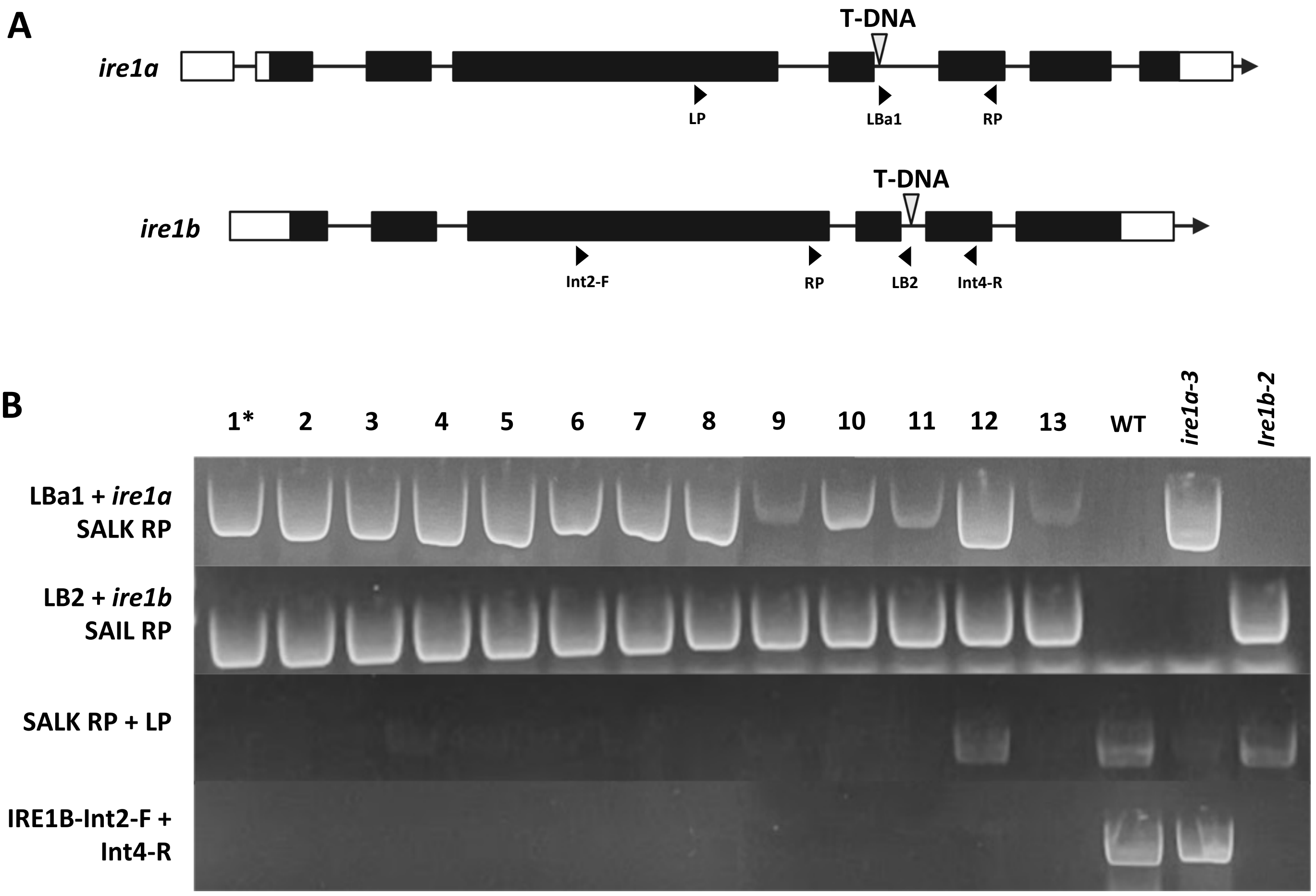

Supplemental Fig. S10 (A) Schematic maps for IRE1A and IRE1B genes, including T-DNA insertion sites. The black rectangles indicate exons, white regions represent the 5' and 3' UTR regions, and lines denote introns. T-DNA insertion sites in the mutant alleles are marked by the gray arrowhead in each gene. **(B)** PCR characterization and verification of the double mutant *ire1a-ire1b* relative to wild type (WT) and the single mutants (*ire1a-3* and *ire1b-2*) using genomic DNA from *Arabidopsis* seedlings separated on 0.8% agarose gels. Thirteen putative double mutants were genotyped and individual 1 (*) was selected for downstream analyses. Gene-specific primers were used to confirm the presence of the insertion among individuals containing the *ire1a-3* (SALK_002316) and the *ire1b-2* (SAIL_238_F07) alleles (bottom two rows: SALK RP + LP and IRE1B-Int2-F + Int4-R, respectively). The presence of the T-DNA alleles were also confirmed using T-DNA specific primers (top two rows: LBa1 + *ire1a* SALK RP and LB2 + *ire1b* SAIL RP).
